# STAT3 activates MSK1-mediated histone H3 phosphorylation to promote NFAT signaling in gastric carcinogenesis

**DOI:** 10.1038/s41389-020-0195-2

**Published:** 2020-02-10

**Authors:** Hongyan Qi, Zhiyi Yang, Chujun Dai, Runan Wang, Xinxin Ke, Shuilian Zhang, Xueping Xiang, Kailin Chen, Chen Li, Jindan Luo, Jimin Shao, Jing Shen

**Affiliations:** 1 Department of Pathology and Pathophysiology, and Department of Radiation Oncology of the Second Affiliated Hospital, Zhejiang University School of Medicine, Hangzhou, 310058 China; 2 Department of Pathology and Pathophysiology, and Department of Medical Oncology of the Second Affiliated Hospital, Zhejiang University School of Medicine, Hangzhou, 310058 China; 3 Department of Pathology, the Second Affiliated Hospital, Zhejiang University School of Medicine, Hangzhou, 310058 China; 4 Institute of Genetics and Department of Genetics, Zhejiang University School of Medicine, Hangzhou, 310058 China; 5 The First Affiliated Hospital, Zhejiang University School of Medicine, Hangzhou, 310058 China; 6 Department of Pathology and Pathophysiology, and Cancer Institute of the Second Affiliated Hospital, Zhejiang University School of Medicine, Hangzhou, 310058 China

**Keywords:** Gastric cancer, Epigenetics

## Abstract

Epigenetic abnormalities contribute significantly to the development and progression of gastric cancer. However, the underlying regulatory networks from oncogenic signaling pathway to epigenetic dysregulation remain largely unclear. Here we showed that STAT3 signaling, one of the critical links between inflammation and cancer, acted as a control pathway in gastric carcinogenesis. STAT3 aberrantly transactivates the epigenetic kinase mitogen- and stress-activated protein kinase 1 (MSK1), thereby phosphorylating histone H3 serine10 (H3S10) and STAT3 itself during carcinogen-induced gastric tumorigenesis. We further identified the calcium pathway transcription factor NFATc2 as a novel downstream target of the STAT3-MSK1 positive-regulating loop. STAT3 forms a functional complex with MSK1 at the promoter of *NFATc2* to promote its transcription in a H3S10 phosphorylation-dependent way, thus affecting NFATc2-related inflammatory pathways in gastric carcinogenesis. Inhibiting the STAT3/MSK1/NFATc2 signaling axis significantly suppressed gastric cancer cell proliferation and xenograft tumor growth, which provides a potential novel approach for gastric carcinogenesis intervention by regulating aberrant epigenetic and transcriptional mechanisms.

## Introduction

Gastric cancer (GC) is currently the third leading cause of cancer death worldwide with high prevalence in many Eastern Asia countries, particularly in China, Japan, and South Korea^1,2^. *Helicobacter pylori* (*H. pylori*) infection is the main risk factor for GC. However, other environmental factors, including diet, smoking, and alcohol consumption are also likely of major importance. Although genetic abnormalities function importantly in GC development (for example, germline mutation of the E‑cadherin gene in familial patient), epigenetic alterations are now widely recognized to be involved in gastric tumorigenesis^3,4^. Accumulating evidence suggests that epigenetic abnormalities, especially DNA methylation and histone modifications, promote carcinogenesis through active mechanisms in both gastric tumors and pre-malignant lesions. For instance, *H. pylori*-induced chronic inflammation leads to aberrant DNA methylation, which results in the inactivation of key cancer-related genes in gastric mucosa^3^. Alterations of multiple histone modifications (methylation of histone H3 at K36, K4, and K27) also have been found at distinct regulatory elements controlling the transcription of oncogenes and tumor suppressors in GC^5,6^. However, despite plenty of research findings, it is still largely unclear when and how the initial aberrant changes of epigenetic modifications take place in gastric epithelial cells.

Among multiple gastric carcinogens, *N*-nitroso compounds (NOCs), either produced endogenously and increased upon *H. pylori* infection or exposed exogenously from diet, have shown strong carcinogenic effects in animal studies. Individuals with a high-level exposure to NOCs are also hypothesized to be at increased risk of developing gastrointestinal cancer^7,8^. We and other groups have reported that various cellular signaling pathways such as proliferation, DNA repair, oxidative stress and inflammatory pathways^9–15^, as well as epigenetic remodeling regulations act as crucial players in *H. pylori* and NOC-induced carcinogenesis^3,16^. We also revealed that, upon NOC treatment, the dysregulation of histone modifications occurred as part of DNA damage responses and contributed to numerous cancer-related gene expression changes^17^. These findings prompted us to speculate that critical signaling pathways activated by carcinogens may interplay with epigenetic changes, thereby contributing to gastric carcinogen-induced carcinogenesis.

Hitherto, few pathways have been reported to directly cooperate with epigenetic aberrations, and the underlying mechanisms remain elusive. Among them, signal transducer and activator of transcription 3 (STAT3) signaling pathway is a prominent one. As a well-known player linking inflammation and cancer, STAT3 is frequently overexpressed in tumor cells or tissues, including GC^18–20^. By regulating the expression of numerous oncogenic genes, STAT3 promotes the development of different types of cancer. Through epigenetic mechanisms, activated STAT3 has been reported to modulate the expression of DNA methyltransferases and histone methylases or demethylases in hematological malignancies and solid tumors^21–23^. A regulatory complex was also identified between STAT3 and those chromatin-associated enzymes in epigenetic silencing or activation of vital tumor-promoting genes^24–27^.

To explore the possible interplay between STAT3 signaling and epigenetic alterations in gastric cancer development, in the current study, we investigated the epigenetic changes of aberrant histone modifications and the involvement of STAT3 pathways in gastric carcinogenesis. We demonstrated that STAT3 signaling was highly activated during *H. pylori* plus NOC-induced murine gastric tumorigenesis and human gastric cell malignant transformation, in parallel with an augment of histone H3 Ser10 phosphorylation. Further studies revealed that STAT3 activated the transcription and function of mitogen- and stress-activated protein kinase 1 (MSK1), which phosphorylated H3 and promoted the downstream inflammatory signaling during gastric carcinogenesis.

## Results

### STAT3 contributes to enhanced H3S10 phosphorylation in gastric carcinogenesis

**Fig. 1:**
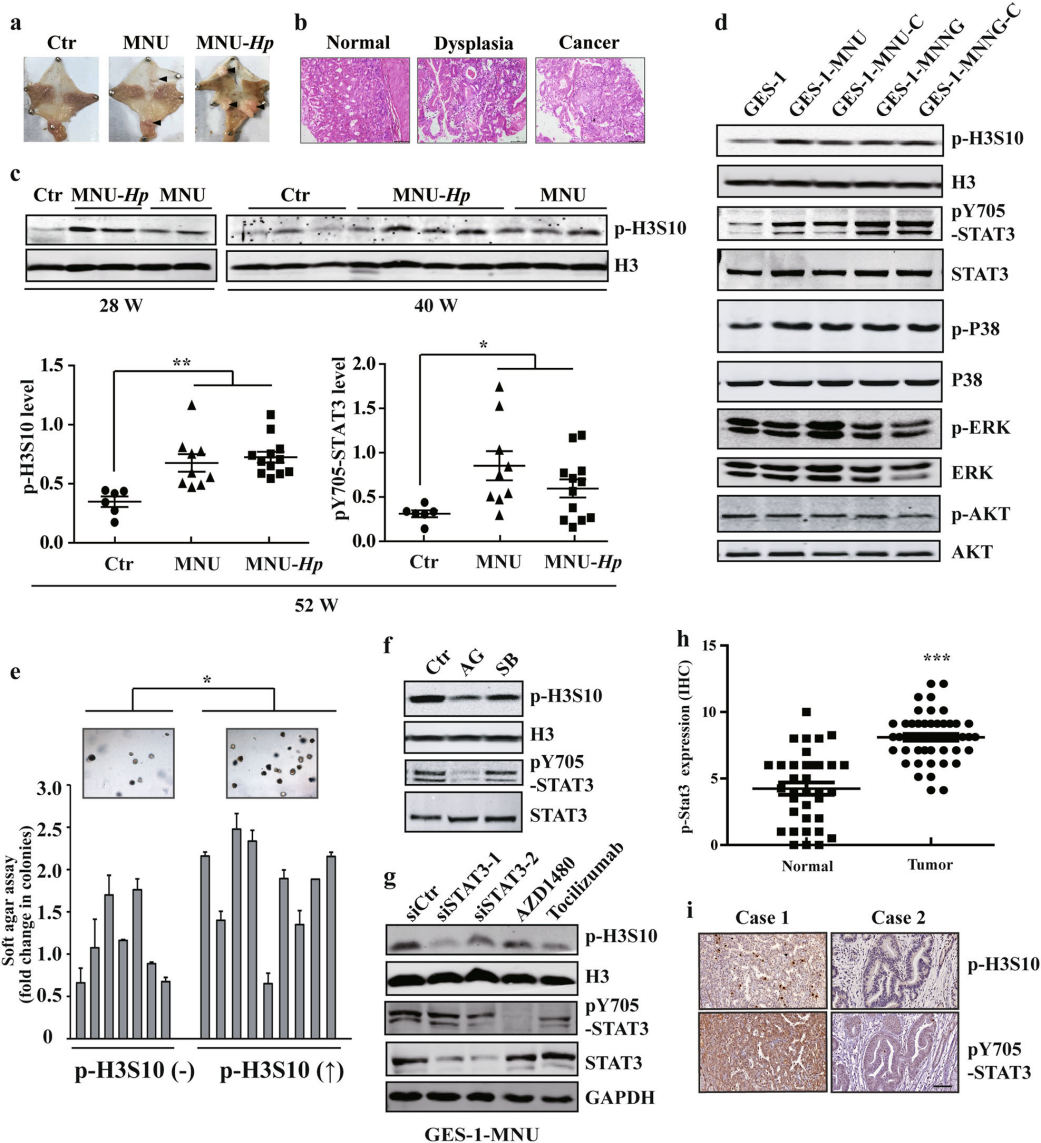
STAT3 contributes to enhanced H3S10 phosphorylation in gastric carcinogenesis. **a** Representative image of NOC (*N*-methyl-*N*-nitroso-urea, MNU) and *H. pylori* plus NOC -induced mouse model of gastric cancer. **b** HE staining of stomach tissues from the carcinogen-treated mice. **c** Histone H3 phosphorylation was analyzed by WB of stomach tissues from the carcinogen-treated mice at different times. **d** WB analysis of signaling pathways related to cell proliferation in GES-1 and NOC-treated cells with the indicated antibodies. **e** Cell colony-forming ability in soft agar of p-H3S10 increased or unchanged subcloned NOC-treated cells. **f**, **g** AG490 50 μM, SB203580 10 μM, AZD1480 2 μM, Tocilizumab 10 μM, scramble siRNA (siCtr) or STAT3 siRNA were used to treat the MNU-transformed cells, respectively. The level of p-H3S10 was determined by WB. **h** STAT3 expression score by IHC in 52 paired gastric cancer tissues. **i** The representative images of STAT3 and p-H3S10 levels in human gastric cancer tissues by IHC. Scale bar: 200μm. The analyses were repeated three times, and the results were expressed as mean ± SD. *^/#^*p* < 0.05, **p < 0.01, ***p < 0.001. GES-1-MNNG or GES-1-MNU: MNNG- or MNU-induced malignantly transformed GES-1 cell; GES-1-MNNG-C or GES-1-MNU-C: subcloned cells derived from GES-1-MNNG or GES-1-MNU.

To explore the epigenetic alterations during gastric carcinogenesis, we first analyzed the well-established *H. pylori* plus NOC (*N*-methyl-*N*-nitroso-urea, MNU)-induced mouse model of GC^28^. Fifty-two weeks after carcinogen treatment, mice were sacrificed and stomach tissues were subject to histologic assessment (Fig. 1a, b). As expected, all the mice in the control group (*n* = 6) had normal histology, whereas 5 of 9 (55.6%) and 9 of 12 (75%) mice developed preneoplastic or neoplastic lesions in MNU alone and *H. pylori* plus MNU group, respectively (Supplementary Fig. 1a). Then we attempt to determine the possible epigenetic changes during the progression of gastric carcinogenesis in the mouse model. Notably, our results revealed that *H. pylori* infection in MNU-treated mice strongly induced histone H3 phosphorylation at Ser10 (p-H3S10) as early as 28 weeks after carcinogen exposure (Fig. 1c). MNU treatment, although weaker than *H. pylori* plus MNU group, also showed a significant p-H3S10 increase compared with the control group. Meanwhile, we detected an accompanying significant activation of STAT3 in carcinogen-treated murine gastric tissues in both MNU only and *H. pylori* plus MNU groups.

To determine the relationship and underlying mechanisms between aberrant H3 phosphorylation and STAT3 pathway, we further established a cell malignant transformation model with the chronic exposures of human gastric epithelial GES-1 cells to the NOC carcinogens, MNU and *N*-methyl-*N*′-nitro-*N*-nitrosoguanidine (MNNG). NOC-treated cells showed an increase of colony formation rate and an ability of tumor formation in nude mice (Supplementary Fig. 1b, c). We demonstrated that NOC exposure strongly induced histone H3 phosphorylation at Ser10 (p-H3S10) and Ser28 (p-H3S28) without altering the cell cycle distribution (Fig. 1d and Supplementary Fig. 1d, e). However, only the augment of p-H3S10 expression correlated with the increased colony-forming ability of malignant transformed cell subclones (Fig. 1e). Then we analyzed the activation of STAT3 and other cancer-related pathways. The results showed that STAT3 and p38-MAPK pathways were highly activated, whereas no significant changes were found for other pathways such as ERK and AKT signaling (Fig. 1d). Further studies using specific inhibitors revealed that inhibition of STAT3 signaling triggered a remarkable reduction of p-H3S10, while p38-MAPK inhibition only caused a slight decline in this modification (Fig. 1f). STAT3 siRNA knockdown or applying anti-interleukin-6 receptor antibody (Tocilizumab) also confirmed the regulatory effect of STAT3 pathway on p-H3S10 enhancement (Fig. 1g, Supplementary Fig. 1f). Moreover, STAT3 pathway was found highly activated in both NOC-transformed cell subclones and clinical gastric cancer tissues, coordinating with the elevation of H3S10 phosphorylation (Supplementary Fig. 1g, Fig. 1h, i). Collectively, these results determine an aberrantly enhanced H3S10 phosphorylation in gastric carcinogenesis and support a notion that STAT3 signaling acts as an upstream regulator of this modification.

### MSK1 phosphorylates H3S10 during carcinogen-induced transformation and promotes gastric cancer cell proliferation

**Fig. 2:**
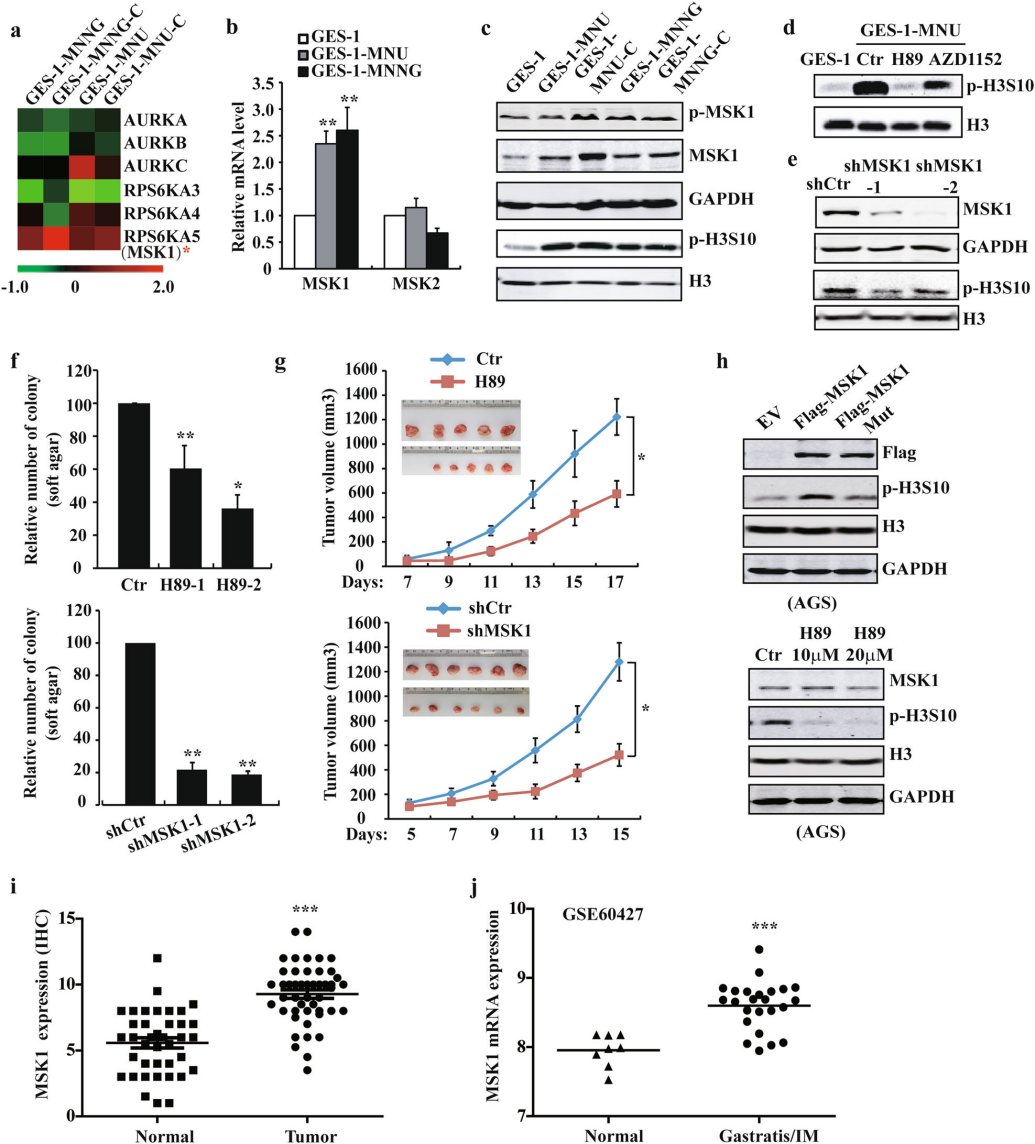
MSK1 phosphorylates H3S10 during carcinogen-induced transformation and is crucial for gastric cancer cell proliferation. **a** The heatmap of RNA-seq analysis of H3S10 kinases in GES-1 and NOC-transformed cells. **b** RT-qPCR analysis of the mRNA expression of MSKs in NOC-transformed cells. **c** MSK1 phosphorylation and total protein, p-H3S10 levels in NOC-transformed cells detected by WB analysis. **d** p-H3S10 level was determined by WB after H89 10 μM, AZD1152 treatment for 24 h or **e** MSK1 stably silenced in NOC-transformed cells. **f** Soft agar and **g** xenograft assay (*n* = 6) were performed after H89 treatment or MSK1 stably silenced in NOC-transformed cells cells. **h** p-H3S10 level was determined by WB after overexpression of MSK1 wild-type and inactive mutant (Mut), or treated with H89 in AGS cells. **i** MSK1 expression score in 52 paired gastric cancer tissues by IHC. **j** Analysis of MSK1 expression in GEO dataset (GSE60427). The analyses were repeated three times, and the results were expressed as mean ± SD. *p < 0.05, **p < 0.01, ***p < 0.001.

To study the kinase(s) responsible for enhanced p-H3S10 during carcinogen exposure, we investigated the expression profile of kinases for H3S10 in NOC-transformed cells. Among them, the mitogen- and stress-activated protein kinase-1 (MSK1) was observed dramatically upregulated and activated in the transformed cells, whereas the close homolog MSK2 and mitotic kinase Aurora B remained unaffected (Fig. 2a–c, Supplementary Fig. 2a). Dynamic expression studies revealed that MSK1 increased as early as 1 week post treatment (Supplementary Fig. 2b). Inhibition of MSK1 with inhibitor H89 or specific siRNA substantially abrogated the upregulation of p-H3S10 level (Fig. 2d and Supplementary Fig. 2c). For functional studies, MSK1 was stably silenced by shRNA sequences. MSK1 knockdown significantly decreased colony formation of transformed cells and reduced tumor sizes in subcutaneous xenograft models with a remarkably attenuated level of p-H3S10 (Fig. 2e–g, and Supplementary Fig. 2d–f). Similar results were obtained in H89-mediated MSK1 inhibition experiments, supporting that MSK1 kinase is predominantly responsible for NOC-induced phosphorylation of H3S10.

Then we sought to assess the role of MSK1/p-H3S10 pathway in GC cells. The observed increase of p-H3S10 after MSK1 overexpression in AGS cells, and the decrease of p-H3S10 after H89-mediated MSK1 inhibition in AGS and MKN45 cell lines confirmed MSK1-controlled phosphorylation of H3S10 in GC cells (Fig. 2h and Supplementary Fig. 2g). H89 treatment also robustly inhibited the anchorage-independent growth of GC cells (Supplementary Fig. 2h). Furthermore, IHC analysis of clinical gastric cancer tissues showed a significant increase of MSK1 level in tumors compared to normal tissues (Fig. 2i and Supplementary Fig. 2i). Interestingly, in another publicly available dataset (GSE60427)^29^, we found that MSK1 was upregulated in gastritis/intestinal metaplasia (IM) group which was *H. pylori* positive (Fig. 2j), supporting the involvement of MSK1 activation in the early stage of gastric tumor development.

### STAT3 induces transcriptional activation of MSK1 and forms a positive feedback loop with MSK1

**Fig. 3:**
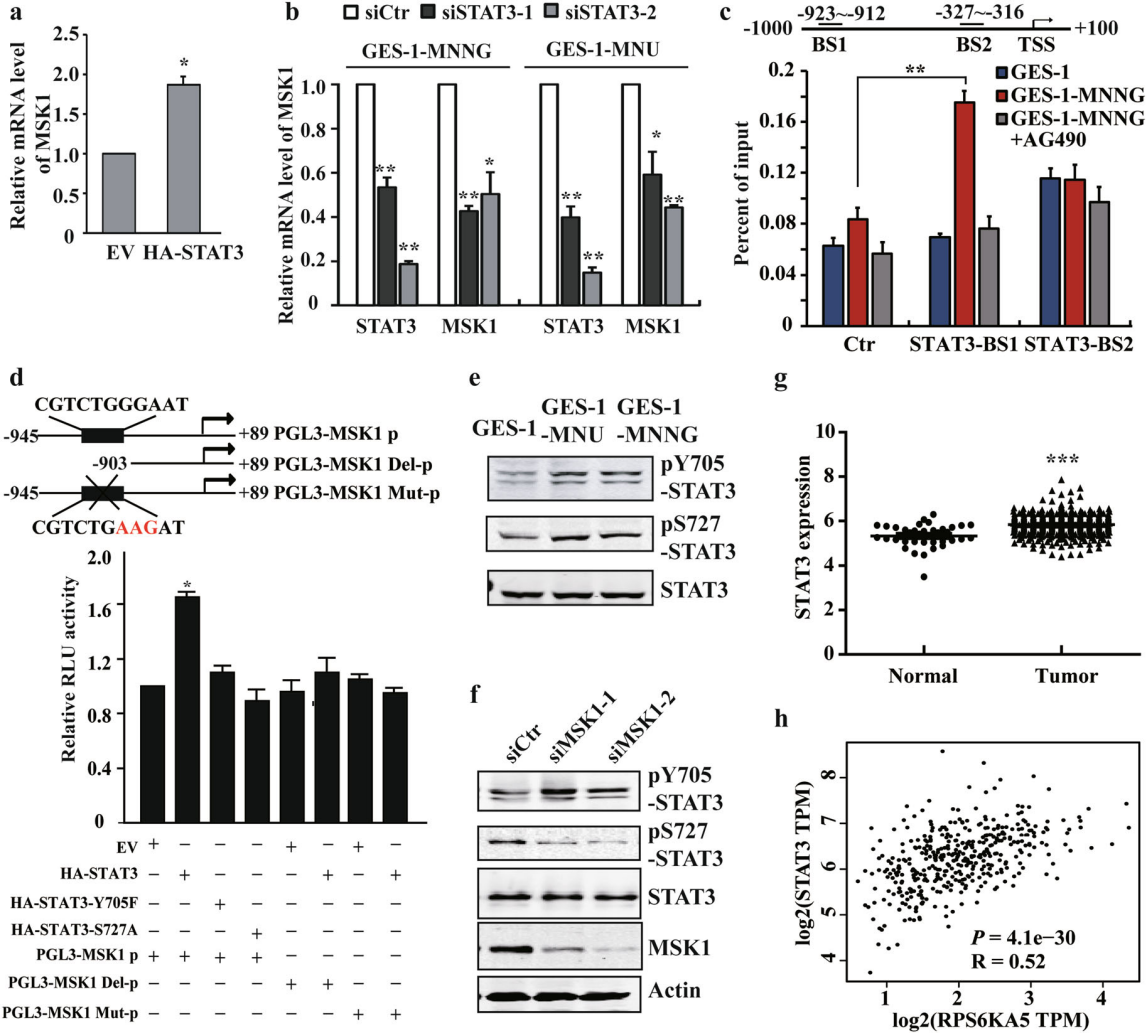
STAT3 induces transcriptional activation of MSK1 and forms a positive feedback loop with MSK1. **a**, **b** RT-PCR analysis of MSK1 expression after STAT3 overexpression or siRNA knockdown in GES-1 or NOC-transformed cells. **c** NOC-transformed cells were treated with vehicle or AG490. The binding of STAT3 in the specific sites of MSK1 promoter were analyzed by ChIP-qPCR with anti-STAT3 antibody. Ctr: control site. BS1: binding site 1. BS2: binding site 2. **d** pLG3-MSK1 promoter constructs and STAT3 full-length or muntants were co-transfected into the cells, and the transcriptional activity of MSK1 promoter was analyzed by the luciferase reporter assay. **e** STAT3 Y705 or S727 phosphorylation levels were examined by WB with the specific antibodies in malignantly transformed cells. **f** STAT3 phosphorylation levels in the transformed cells following MSK1 siRNA knockdown were detected by WB analysis. **g** TCGA database analysis of STAT3 expression. **h** The correlation between STAT3 and MSK1 expression of the TCGA database were generated by GEPIA (Gene Expression Profiling Interactive Analysis, http://gepia.cancer-pku.cn/). The analyses were repeated three times, and the results were expressed as mean ± SD. *p < 0.05, **p < 0.01, ***p < 0.001.

The significance of STAT3 pathway in p-H3S10 modulation and further explored activation of MSK1/p-H3S10 axis prompted us to investigate whether STAT3 exert its functions on histone phosphorylation by directly regulating MSK1. To address this notion, we ectopicly expressed STAT3 in normal GES-1 cells or knocking down STAT3 in NOC-transformed cells. The results showed that overexpression of STAT3 significantly elevated MSK1 expression, whereas silencing STAT3 remarkably decreased the level of MSK1 (Fig. 3a, b and Supplementary Fig. 3a). Moreover, application of JAK/STAT3 pathway inhibitors efficiently blocked MSK1 upregulation after NOC exposure (Supplementary Fig. 3b). Bioinformatic analysis further revealed two potential STAT3-binding sites (positions −923 to −912 and −327 to −316, respectively) located in the MSK1 promoter region. Using chromatin immunoprecipitation (ChIP), we detected a significant increase in STAT3 recruitment to the distal rather than the proximal STAT3 site, which could be further abolished by the STAT3 pathway inhibitor AG490 (Fig. 3c). In addition, luciferase reporter assay confirmed that mutation or deletion of the distal site almost abrogated MSK1 promoter-driven transcription (Fig. 3d). These data illustrate that STAT3 is a direct transcription regulator of MSK1 upon NOC treatment.

It has been reported that MSK1 regulates downstream gene transcription both through modulating histone phosphorylation and transcription factor activation^30–33^. STAT3 serine 727 (Ser727) was also reported as a direct substrate of MSK1 in response to UV stress^34^. In accordance, besides the canonical Tyr705 site, we found that STAT3 was phosphorylated at Ser727 in NOC-transformed cells (Fig. 3e). STAT3 mutants Y705F and S727A remarkably reduced MSK1 reporter activity, supporting the critical role of both residues in MSK1 transactivation (Fig. 3d). Intriguingly, blocking MSK1 activation by inhibitor H89 or MSK1 knockdown significantly decreased the phosphorylated Ser727 level of STAT3 (Fig. 3f and Supplementary Fig. 3c). Further analysis of the cancer genome atlas (TCGA) database identified a significant upregulation of STAT3 in gastric cancer tissues, and a positive correlation between the expression of STAT3 and MSK1 (Fig. 3g, h).

Taken together, these results support that STAT3 directly transactivates MSK1 during carcinogen-induced malignant transformation, and vice versa, MSK1 modulates the activation of STAT3 by phosphorylating its Ser727 residue, thus forming a positive feedback loop between STAT3 and MSK1 in gastric carcinogenesis.

### NFATc2 is a downstream target of MSK1-mediated H3S10 phosphorylation

**Fig. 4:**
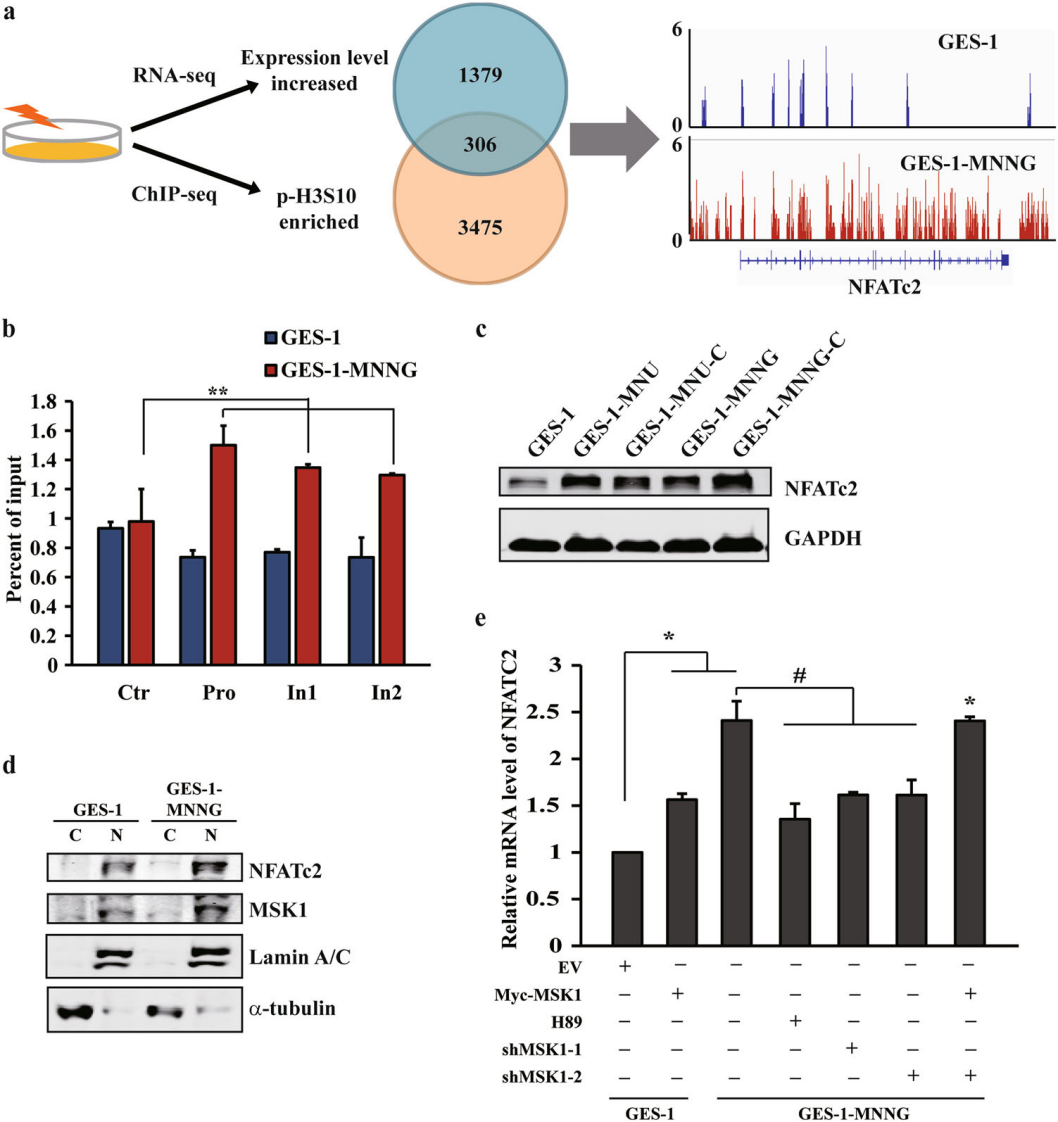
NFATc2 is a downstream target of MSK1-mediated H3S10 phosphorylation. **a** The overlap of genes with increased expresion in RNA-seq (blue) and enriched p-H3S10 signals in ChIP-seq (orange) results in NOC-transformed cells compared with GES-1 cells. The snapshop of p-H3S10 ChIP-Seq signals at the *NFATC2* gene locus in NOC-transformed and GES-1 cells. **b** ChIP–qPCR of p-H3S10 signals in NOC-transformed and GES-1 cells using the indicated primer pairs. Pro: promoter site. In: intron site. **c**, **d** NFATc2 total and nuclear protein levels were detected by WB in GES-1 and NOC-transformed cells. **e** NFATc2 mRNA expression in GES-1 and NOC-transformed cells with MSK1 overexpression, H89 treatment or MSK1 knockdown. The analyses were repeated three times, and the results were expressed as mean ± SD. *^/#^*p* < 0.05 and **p < 0.01.

Histone H3 phosphorylation has been linked to transcriptional activation of genes in response to multiple extracellular signals^33,35,36^. To determine the relationship between carcinogen-induced p-H3S10 enrichment and gene regulation, RNA-seq analysis of NOC-transformed cells were firstly investigated. As expected, Kyoto Encyclopedia of Genes and Genomes (KEGG) analysis indicated that “pathways in cancer” is one of the most significantly altered gene set concepts in NOC-transformed cells, and gene set enrichment analysis (GSEA) revealed a large fraction of IL6/STAT3 downstream genes that displayed significant alterations (Supplementary Fig. 4a, b). We further analyzed the ChIP-seq profiles of H3S10 phosphorylation, and identified 3475 genes associated with the increased p-H3S10 mark in the transformed cells. Although the H3 phosphorylation signal preferentially enriched in intragenic regions on a genome-wide scale, the differential peaks were more obvious within the promoter and throughout gene bodies (Supplementary Fig. 4c, d). After combined the ChIP-seq profiles with the RNA-seq data, we found that 306 genes might be directly regulated by p-H3S10, as they showed an increase in both p-H3S10 modification and expression level in the transformed cells (Fig. 4a). Out of them, 30 genes involved in pathways, such as metabolic, tight junction and mRNA surveillance pathways, got highly increased ChIP-seq signals (binding regions > 2, change fold > 2) and peaks mapping to exon or transcription start site (TSS) ± 2 kb regions (Supplementary Table 1).

Among them, calcineurin/nuclear factor of activated T cells (NFAT) family member NFATc2 is one of the genes enriched abundant p-H3S10 signals (Supplementary Table 1 and Fig. 4a), and ChIP-qPCR examination further confirmed the increased p-H3S10 in the promoter and intron regions of NFATc2 (Fig. 4b). Consistently, the total and nuclear protein level of NFATc2 was also found substantially upregulated in transformed cells and GC cells (Fig. 4c, d and Supplementary Fig. 4e). Ectopic expression of MSK1 in GES-1 cells was sufficient to induce the production of NFATc2, whereas inhibition or knockdown of MSK1 in transformed cells or GC cells resulted in a significant reduction of NFATc2, which can be reversed by MSK1 rescue (Fig. 4e and Supplementary Fig. 4f). These results support that NFATc2 is a direct downstream target of MSK1-mediated H3S10 phosphorylation following NOC carcinogen treatment.

### NFATc2 contributes to gastric carcinogenesis by affecting the inflammatory pathways

**Fig. 5:**
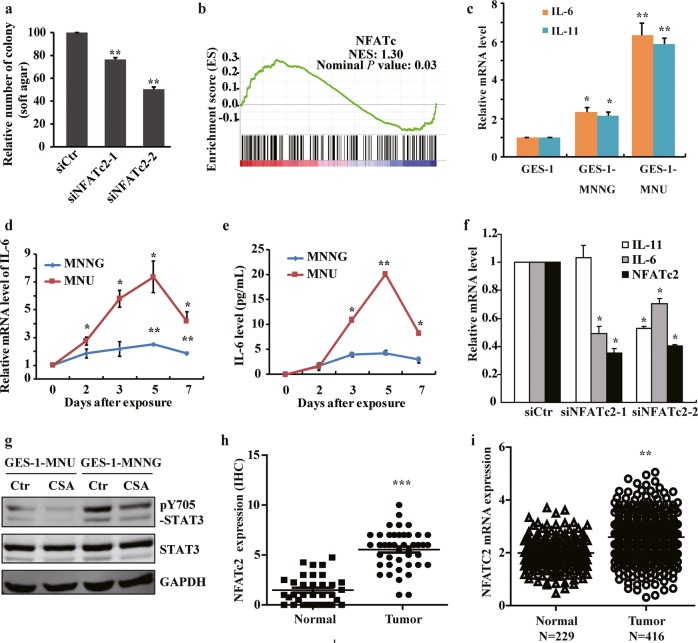
NFATc2 contributes to gastric carcinogenesis by affecting the inflammatory pathways. **a** Cell anchorage-independent growth in transformed cells with NFATC2 siRNA knockdown. **b** Gene set enrichment plots of differentially expressed genes belonging to the NFATc pathway in NOC-treated cells. *P* value is determined by GSEA software. **c** IL-6 and IL-11 mRNA expression in malignant transformed cells by RT-qPCR. **d** IL-6 expression by PCR after NOC treatment at different times. **e** IL-6 production detected by ELISA after NOC treatment at different times. **f** RT-qPCR analysis of IL-6 and IL-11 mRNA levels after knockdown NFATc2 by siRNA. **g** The transformed cells were treated by cyclosporine A (CSA) 10 μM for 24 h, and STAT3 Y705 phosphorylation was detected by WB. **h** and **i** NFATc2 expression in 52 paired gastric cancer tissues by IHC, and TCGA database analysis, respectively. The analyses were repeated three times, and the results were expressed as mean ± SD. *p < 0.05, **p < 0.01, ***p < 0.001.

NFAT transcription factors can be induced by inflammatory conditions, thereby contributing to malignant properties including cell invasion, migration, survival, proliferation, and tumor stemness^37,38^. To further clarify the functional importance of NFATc2 in gastric carcinogenesis, we depleted NFATc2 expression and found a reduced anchorage-independent growth in NOC-transformed cells (Fig. 5a). GSEA analysis also revealed a significant enrichment of NFATC downstream genes in transformed cells (Fig. 5b). It has been reported that NFATc2 plays a critical role in inflammation-associated colorectal tumorigenesis by modulating the production of proinflammatory cytokine IL-6^39^. Interestingly, besides the activation of STAT3 protein, we also observed a remarkable induction of cytokines IL-6 and IL-11 in NOC-transformed cells (Fig. 5c). Further analysis revealed that IL-6 and IL-11 were induced rapidly after exposing to NOC, and reached a peak at 5 days post treatments (Fig. 5d, e and Supplementary Fig. 5a). Inhibiting NFATc2, however, substantially decreased the cytokine expression, followed by the STAT3 pathway suppression (Fig. 5f, g), indicating that NFATc2 affected carcinogen-induced transformation at least partially by contributing to the control of proinflammatory cytokine production.

Then we examined clinical gastric specimens for NFATc2 and IL-6 expression. IHC staining results showed that NFATc2 significantly upregulated in tumors compared with the adjacent normal counterparts, which was in line with the data from TCGA database analysis (Fig. 5h, i). High level activated NFATc2 expression with widespread nuclear and/or cytoplasmic staining were detected in isolated or small clusters of tumor cells, accompanied by an elevated IL-6 cytoplasmic staining in the corresponding areas (Supplementary Fig. 5b). Overall, these results suggest that NFATc2 represents as an important functional target gene of MSK1-mediated H3S10 phosphorylation by connecting with the inflammatory pathways in gastric carcinogenesis.

### MSK1 and STAT3 can be recruited to the promoter of *NFATC2*, and activate its expression by coupling histone phosphorylation

**Fig. 6:**
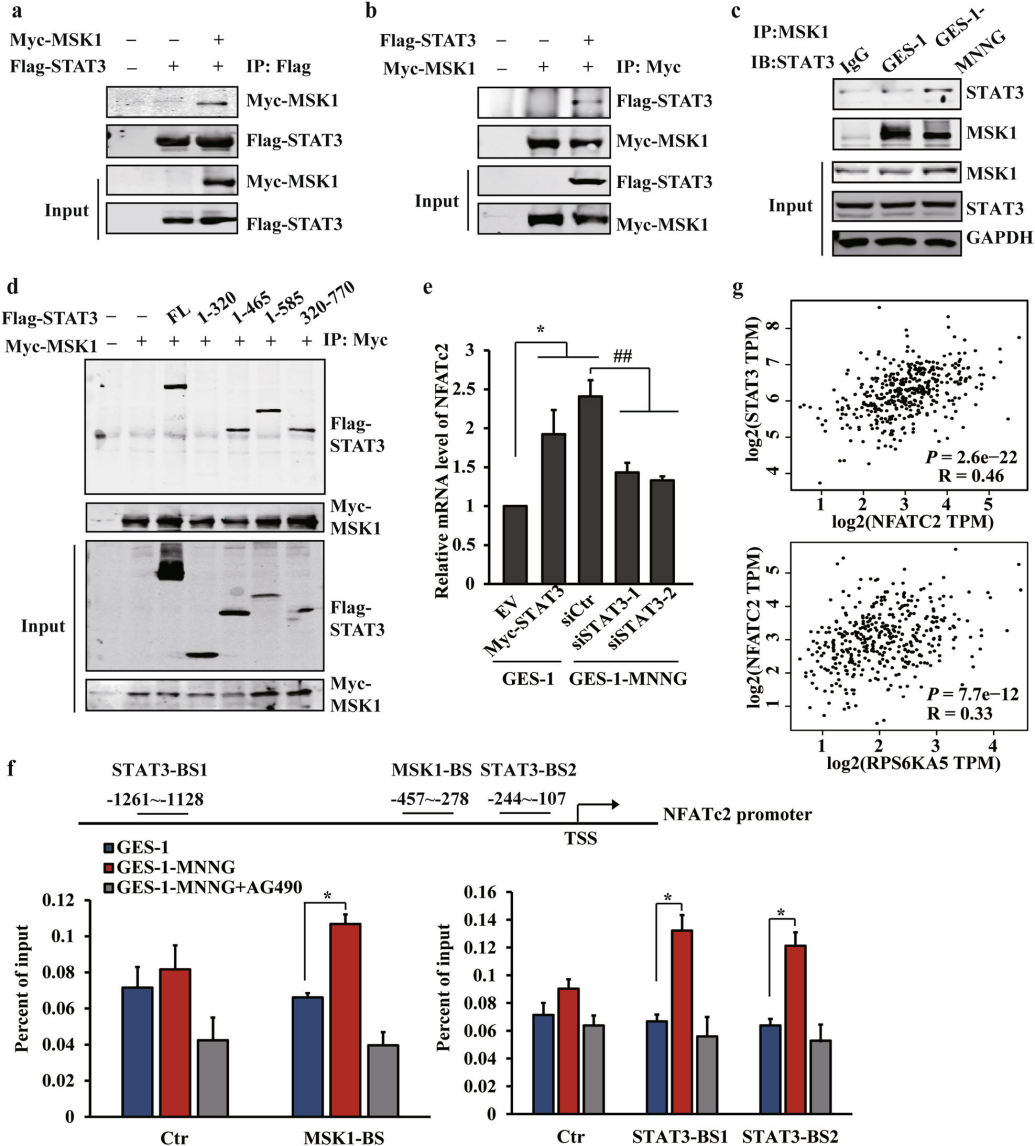
MSK1 and STAT3 can be recruited to the promoter of NFATC2, and activate its expression by coupling histone phosphorylation. **a**, **b** STAT3 and MSK1 protein interaction was derived from HEK 293T cells following the transfection with Myc-MSK1 and Flag-STAT3 by immunoprecipitation assay (IP). **c** Endogenous interaction between Stat3 and MSK1 by IP analysis derived from GES-1 or MNNG-transformed cells. **d** Flag-STAT3 fragments were co-expressed with Myc-MSK1 wild-type in HEK293T cells. After anti-Myc immunoprecipitation, coprecipitated STAT3 was revealed by immunoblotting. **e** RT-qPCR analysis of NFATc2 mRNA expression after STAT3 overexpression or siRNA knockdown. **f** The binding of MSK1 (left) or STAT3 (right) to the promoter of *NFATc2* was analyzed by ChIP assay with anti-MSK1 or anti-STAT3 antibody in the MNNG-transformed cells treated with vehicle or AG490. Ctr: control site. MSK1-BS: MSK1 binding site. STAT3-BS1: STAT3 binding site 1. STAT3-BS2: STAT3 binding site 2. **g** The correlation between STAT3 and NFATc2 expression or MSK1 and NFATc2 expression of the TCGA database were analyzed by GEPIA. The analyses were repeated three times, and the results were expressed as mean ± SD. *p < 0.05 and ^##^*p* < 0.01.

Since MSK1 phosphorylated both histone H3 and the transcription factor STAT3 in carcinogen-induced transformation, we reasoned that MSK1 and STAT3 might cooperate with each other to regulate chromatin status, thereby altering gene transcription. Using immunoprecipitation analysis, we demonstrated that ectopically coexpressed Myc-tagged MSK1 and Flag-tagged STAT3 associated with each other (Fig. 6a, b). We also detected an increased interaction between endogenous MSK1 and STAT3 in NOC-transformed cells (Fig. 6c). By using the different core fragments of STAT3, we further demonstrated that the DNA-binding domain (amino acids 320–465) of STAT3 was crucial for the MSK1-STAT3 interaction (Fig. 6d). Accordingly, ectopic expression of STAT3 in GES-1 cells triggered a significant upregulation of NFATc2, whereas knockdown of STAT3 in NFATc2-elevated transformed cells or GC cells resulted in a robust reduction of this gene (Fig. 6e and Supplementary Fig. 4f).

Then we attempted to determine whether MSK1 and STAT3 can be co-recruited and directly regulate NFATc2 transcription. As expected, ChIP analysis revealed that MSK1 enriched at the same regions as H3S10 phosphorylation, especially the promoter of *NFATC2* (Fig. 6f). Interestingly, when we performed in silico prediction of putative STAT3-binding sites in the promoter of *NFATc2*, two potential sites were successfully identified not at but surrounding the MSK1/p-H3S10 enriched region, which was further confirmed by ChIP-qPCR analysis (Fig. 6f). Notably, the inhibitor of STAT3 pathway dramatically reduced the enrichment of both STAT3 and MSK1, indicating that MSK1 regulates the phosphorylation of H3S10 in a STAT3-dependent manner (Fig. 6f). Likewise, the direct regulation of NFATc2 expression by STAT3 and MSK1 was strengthened in patients; NFATc2 expression positively correlated with both STAT3 and MSK1 transcripts in patient specimens according to the TCGA database (Fig. 6g). Altogether, these data suggest that STAT3 signaling and MSK1/p-H3S10 pathway collaborate to modulate the production of NFATc2 in gastric carcinogenesis.

### STAT3/MSK1/NFATc2 axis is activated in carcinogen-induced gastric tumorigenesis and correlates with poor prognosis in patients with gastric cancer

**Fig. 7:**
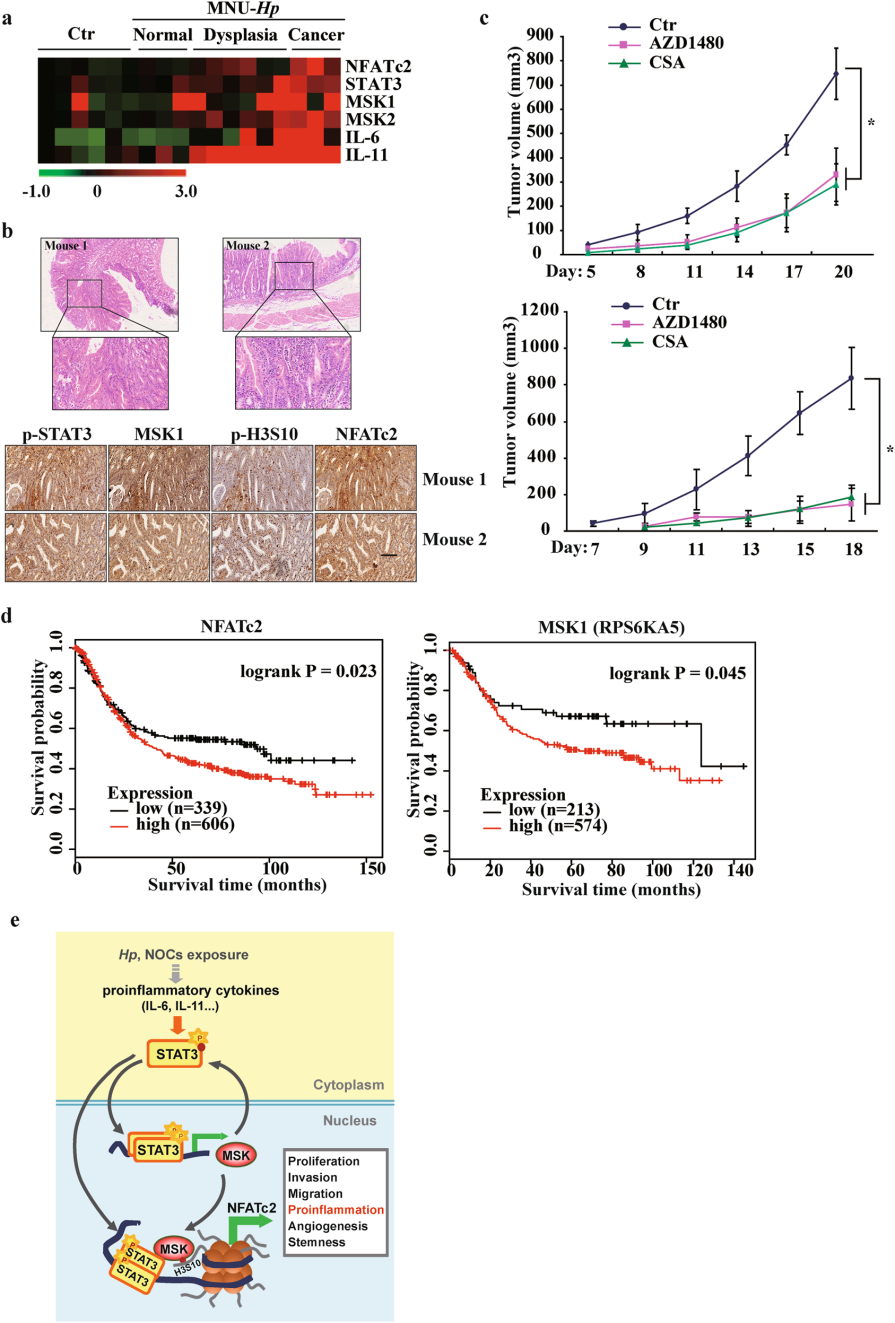
STAT3/MSK1/NFATc2 axis is activated in carcinogen-induced gastric tumorigenesis and correlates with poor prognosis in patients with gastric cancer. **a** Heatmap of the mRNA levels of NFATc2, STAT3, MSK1, IL-6 and IL-11 in stomach tissues from mice treated with or without carcinogens. **b** The representative images of HE staining, p-STAT3 Y705, MSK1, p-H3S10, NFATc2 expression of stomach tissues from the mice treated with carcinogens by IHC. Scale bar: 200 μm. **c** The tumor volume of xenograft assay (*n* = 8, for each group) in NOC-transformed cell and gastric cancer cell MKN45 following the inhibition of STAT3 or NFATc2 by AZD1480 or CSA, respectively. The results were expressed as mean ± SD. *p < 0.05. **d** Kaplan–Meier survival curves by log-rank tests on gastric cancer patients stratified by MSK1 and NFATc2 expression levels for overall survival. **e** Schematic map of Stat3/MSK1/NFATc2 activation model after carcinogens exposure.

We further analyzed the role of STAT3/MSK1/NFATc2 axis in *H. pylori* plus NOC-induced mouse model of GC. RNA-seq analysis revealed that the expression level of STAT3, MSKs, and NFATc2 substantially increased in carcinogen-treated mice compared with control mice, in parallel with a dramatic upregulation of proinflammatory cytokines IL-6 and IL-11 (Fig. 7a). IHC staining results confirmed that the STAT3/MSK1/NFATc2 axis was highly activated in mouse gastric adenocarcinoma samples (Fig. 7b). Treatment with the inhibitors of STAT3 and NFAT pathway, however, significantly reduced xenograft tumor sizes and retarded growth rates of NOC-transformed cells and gastric cancer cells (Fig. 7c and Supplementary Fig. 6a). We then assessed the expression level of the STAT3/MSK1/NFATc2 axis across 31 cancer cohorts available through the TCGA and the Genotype-Tissue Expression (GTEx) dataset. Interestingly, besides gastric cancer, pancreatic adenocarcinoma and brain lower grade glioma also show elevated level for all three genes, and majority cancer types (20/31, 64.5%) present at least one gene significantly upregulated (Supplementary Fig. 6b).

To determine whether the STAT3/MSK1/NFATc2 axis is associated with survival in patients with GC, we analyzed the TCGA data. High NFATc2 expression levels positively correlated with the overall survival (OS) of the patients (Fig. 7d). For MSK1, although the correlation between its expression and adverse OS based on all histologic types did not reach statistical significance, a positive association was revealed in the intestinal-subtype. Collectively, these results support a notion that STAT3/MSK1/NFATc2 form a functional axis in carcinogen-induced gastric tumor development, and provide potential therapeutic targets for human gastric cancer.

## Discussion

It has been reported that epigenetic alterations had a greater impact on cancer risk than genetic alterations in GC^4^. However, the underlying interwoven networks, including upstream signaling pathways, epigenetic modifications, and downstream effector genes remain largely unknown^40,41^. In the current study, we identified STAT3 signaling as the controlling pathway that acts on the transcriptional activation of epigenetic kinase MSK1, thereby contributing to the prominent augment of histone H3S10 phosphorylation during carcinogen-induced gastric tumorigenesis. We also explored that STAT3 forms a functional complex with MSK1 at the promoter of downstream target *NFATc2*, promoting its transcription by coupling histone phosphorylation, thus affecting the inflammatory pathways in gastric carcinogenesis (Fig. 7e).

As the most important oncogenic downstream mediator of the JAK–STAT pathway, deregulated STAT3 has been demonstrated to promote cancer progression mainly through its role as transcription factor. Recent studies illustrated that STAT3 can also affect gene expression via chromatin remodelling, especially epigenetic modifications^42^. STAT3 associates with critical epigenetic modifiers, such as DNA methyltransferase DNMT1 and histone modifiers (EZH2, HAT1, etc.), to facilitate gene silencing or activation^42^. With interaction, histone-modifying enzymes also modify STAT3, including acetylation and methylation changes, which has important consequences for target gene transcription^42^. Interestingly, it was recently reported that STAT3 can directly regulate the transcription of some crucial epigenetic enzymes (DNMT1, EZH2, JMJD3), adding another functional level of STAT3 as an important regulator of global epigenetic modifications and chromatin accessibilities^21–23^. In the present study, we uncovered a novel interplay between STAT3 and the chromatin-associated kinase MSK1 in gastric carcinogenesis: STAT3 drives *MSK1* transcriptional activation and affects numerous gene alterations through histone H3 phosphorylation; MSK1 modulates the activation of STAT3 by phosphorylating its Ser727 residue, thus forming a positive feedback mechanism between these two molecules. Since STAT3-mediated MSK1 upregulation occurred early after carcinogen exposure, our findings suggest that STAT3 incorporates the MSK1 chromatin kinase pathway to promote epigenetic gene reprogramming which may have an important role in carcinogen-induced gastric oncogenesis. Further studies need to determine whether other epigenetic modifiers such as previously reported STAT3-associated DNA methyltransferases and histone modifiers are also incorporated by STAT3 in the initial stage of gastric carcinogenesis.

Moreover, previous studies mainly underline MSK family proteins as the integrator of p38-MAPK and ERK signaling-mediated histone phosphorylation in response to various stimulus^32,43,44^. In the present study, we also found an activation of p38-MAPK but not ERK or AKT signaling after NOC exposure, and inhibiting p38-MAPK pathway resulted in a moderate reduction of p-H3S10. This raise the possibility that p38-MAPK pathway may collaborate with STAT3 signaling to promote the full activation of MSK1 in NOC-induced oncogenesis.

It has been reported that epigenetic remodelling, such as DNA methylation and histone modification changes, contribute to tumorigenesis mainly through affecting the transcriptional output of the genome^3,45^. The presence of p-H3S10 and/or H3S28ph in the promoter regions facilitates histone acetylation and H3K9me2 redistribution, modulates the recruitment of 14-3-3 protein, transcription factors, and chromatin-remodelling complexes, thereby promoting gene transcription^35^. After integrating the ChIP-seq data of p-H3S10 with transcripsome data, we determined that NFATc2, a member belongs to the proinflammatory transcription factor NFAT family, is a major and crucial target gene of MSK1-mediated H3S10 phosphorylation. NFATc2 is initially identified as a critical player in T cell development and function^46^. Recent studies demonstrated that NFATc2 can also be activated and execute oncogenic activity in multiple malignancies, including melanoma, pancreatic and breast cancers^39,47–49^. Besides driving the transcription of tumor-promoting genes such as c-Myc and cyclin-dependent kinase-6 (CDK6), a previous study explored a critical oncogenic role of NFATc2 in colitis-induced colon cancer by control of proinflammatory cytokine IL-6 production^39^. Interestingly, we also found that proinflammatory cytokines (IL-6, IL-11) were rapidly induced after carcinogen exposure and maintained a high level in NOC-transformed and gastric cancer cells. Inhibition of NFATc2 substantially decreased the expression of proinflammatory cytokines, followed by impaired STAT3 activation and suppressed xenograft tumor growth. Thus, our results support NFATc2 signaling as an important link between inflammation and tumorigenesis in gastrointestinal cancers.

Heretofore, excessive activation of proinflammatory cytokines (IL-6, IL-11, TNF-α, etc.) and inflammation-associated signaling pathways, such as STAT3, NF-κB, and p38MAPK, has been linked to gastrointestinal cancer development^50–52^. The aberrantly activated STAT3/MSK1/NFATc2 axis we identified in this study further supports the critical role of the inflammatory response in gastric carcinogenesis. Interestingly, we also revealed the activation of this axis in pancreatic adenocarcinoma and brain lower grade glioma, indicating a similar mechanism may exist in other types of tumor. For the source of proinflammatory cytokines, although the activated myeloid cells are thought to be the main one in the clinical tumor microenvironment, autocrine cytokine signaling in neoplastic epithelial cells are also well-documented^50,53,54^. Accordingly, in MNU-induced mice gastric tumors and human clinical gastric cancer tissues, we observed increased expression of NFATc2 and IL-6 in tumor cells, indicating an autocrine pathway was activated in tumorigenesis. Further investigations will be required to elucidate the underlying mechanisms of tissue inflammation in early gastric carcinogenesis using animal models, as well as clinical samples.

Collectively, in the present study, we provide evidence that STAT3 represents as a nexus that links signaling pathways triggered by carcinogen damage to epigenetic regulation of gene expression via MSK1-mediated H3S10 phosphorylation. The downstream-coupled NFAT signaling further drives the production of proinflammatory cytokines thus contributes to the neoplastic transformation of gastric epithelial cells. Inhibiting STAT3 and NFAT pathway significantly suppressed gastric xenograft tumor growth. These findings shed new light on the STAT3/MSK1/NFATc2 axis as a potential therapeutic target for gastric carcinogenesis intervention by regulating aberrant epigenetic and transcriptional mechanisms.

## Materials and methods

### Cell culture and reagents

Human gastric epithelial GES-1 cell was a gift from China Center for Type Culture Collection, and cultured in DMEM (Invitrogen, CA, USA) with 10% fetal calf serum. Human gastric cancer cell MKN45 and AGS were purchased from the Cell Bank of the Chinese Academy of Sciences (Shanghai, China) and cultured in RPMI 1640 (Invitrogen) and Ham’s F-12K (Kaighn’s) (Invitrogen) with 10% fetal calf serum, respectively. All cell lines were mycoplasma-free and have been authenticated using short tandem repeat profiling within the last three years.

Cells were exposed to 0.5 mmol/L MNU (TRC, Toronto, Canada) or 2 μmol/L MNNG (Sigma, St. Louis, MO, USA) for 2 h in serum free medium. Then the treated medium was removed and cells were recovered in fresh medium at 37 °C. MNU and MNNG exposure was repeated once a week for 4 weeks. After 4 weeks of treatment and 4 weeks of restoration, characteristics related with malignant phenotype were measured. The stimuli concentrations were as follows: H89 (MSK1 inhibitor), 10 μmol/L; AZD1480 (JAK1/2 inhibitor), 2 μmol/L; AG490 (JAK2 inhibitor), 50 μmol/L; SB203680 (p38 MAPK inhibitor), 10 μmol/L. All the compounds were obtained from Selleck (Shanghai, China).

### Immunoblot analysis and immunoprecipitation

Immunoblotting and immunoprecipitation were performed as previously described^52^. Anti-MSK1, anti-Aurora B, anti-β-actin, anti-NFATc2, anti-GADPH, anti-α-tubulin, anti-laminA/C were from Santa Cruz Biotechnology (Santa Cruz, CA, USA). Anti-p-H3S10 and anti-H3 were from Active Motif (Carlsbad, CA, USA). Anti-STAT3, anti-AKT, anti-p42/44, anti-p-STAT3 Y705, anti-p-STAT3 S727, anti-p-AKT S483, anti-p-p42/44 were from Cell Signaling Technology (Danvers, MA, USA).

### RNA analysis and ChIP assay

RNA analysis was performed as previously described^52^ Primers were shown in Supplementary Table 2. For ChIP assays, malignant transformed cells were treated with 50 μmol/L AG490 or mock for 24 h. Sheared chromatin was prepared and incubated with anti-MSK1 and anti-STAT3 antibodys (CST) for collecting the associated DNA. The DNA was quantified by quantitative PCR. Primers for detecting the binding sites in MSK1 promter or NFATc2 promoter/intron were showed in Supplementary Table 2.

### RNA interference (RNAi) and gene knockdown by shRNA

The siRNAs targeting human STAT3 and NFATc2 (GenePharma, Shanghai, China) were transfected into cells using Lipofectamine™ RNAiMAX (Invitrogen) according to the manufacturer’s instruction. A siRNA targeting luciferase was used as negative control. SiRNA sequences were shown in Supplementary table 2.

Knockdown of MSK1 was performed using the lentiviral expression system. The lentiviruses were produced by co-transfecting HEK293T with pLVX-shRNA1 or pLVX-shRNA1-MSK1 shRNA and two packaging plasmids (psPAX2 and pMD2.G). The specific sequences were shown in Supplementary Table 3.

### Anchorage-independent growth assay

The cells (1000 cells) were suspended in culture medium containing 0.4% agarose (Sigma) and seeded onto a base layer of 0.7% agar bed in 6-well plates. After 2 weeks, colonies were stained with crystal violet and photographed. Colonies ≥0.05 mm in diameter were counted.

### Xenograft model

BALB/C male nude mice (4-weeks-old) were subcutaneously injected with 10^6^ transformed cells. Three days after injection, the mice was treated with H89 20 mg/kg by intraperitoneal injection 3 days a week for 2 weeks, or treated with AZD 50 mg/kg by gavage administration 5 days a week for 2 weeks. The long diameter (a) and short diameter (b) of the tumors were measured, and then the volume (V) was calculated using the formula *V* = 1/2 × a × b^2^. Mice were sacrificed and the tumors were obtained, weighted, and histologically examined.

### RNA-seq analysis

Total RNA was isolated from cells either NOC treated or not using TRIzol reagent (Invitrogen). The RNA of each sample was used for RNA-seq and performed by Novogene Corporation. For gene expression analysis, HT Seq v0.6.1 was used to count the read numbers mapped of each gene. Then, RPKM (Reads Per Kilo bases per Million reads) was calculated based on the length of the gene and reads count mapped to this gene. Corrected q-value of 0.05 and log2 (fold change) of one were set as the threshold for significant differential expression.

### IHC

The study was based on a cohort of 52 GC patients at the Second Affiliated Hospital of Zhejiang University School of Medicine and was approved by the ethics committee of the Zhejiang University School of Medicine (2017026). Samples from patients who received preoperative radiation or chemotherapy were excluded. The primary cancer tissues were formalin-fixed and paraffin-embedded for immunohistochemistry. The immunohistochemistry was performed using an Envision Detection System (DAKO, Carpinteria, CA) according to the manufacturer’s instructions. Anti-Ki67 and anti-MSK1 were from Abcam (Cambridge, UK). Anti-NFATc2 was from Santa Cruz Biotechnology. Anti-p-STAT3 Y705 was from CST. The staining results were assessed and confirmed by two independent investigators blinded to the clinical data.

### Dual-luciferase reporter assay

Promoter sequence were synthesized and subcloned into pGL3-Basic vector (Promega, Madison, WI, USA). The mutation (GG-AA) was introduced into pGL3-MSK1 promoter wild-type by site-directed mutagenesis. pGL3-MSK1 promoter constructs were cotransfected with either wild-type or mutants of STAT3 into cells with X-treme GENE HP DNA Transfection Reagent (Roche, Basel, Switzerland). Relative luciferase activity was tested using Dual-Luciferase Reporter Assay System (Promega).

### ChIP-seq

Approximately 5 × 10^7^ cells were collected for each ChIP-seq assay. Chromatin DNAs were precipitated by anti-p-H3S10 (Active Motif) and purified with the Qiagen PCR purification kit. The library construction and sequencing procedures were performed by BGI (Shenzhen, China), following the manufacturer’s instructions (Illumina, San Diego, CA, USA). Clean reads were mapped to the human reference genome hg19 using the SOAP2.21 alignment package, and further analyzed by MACS (Model-based Analysis for ChIP-Seq). Enriched islands for p-H3S10 were identified using SICER with 200 bp window size, 600 bp gap size, and an effective genome size of 80% of the human genome. Peak regions were rendered and visualized using Integrated Genome Viewer (IGV 2.4).

### Animal studies

C57BL/6 female mice (4-weeks-old) were randomly divided into three groups, control, MNU-treated and *H. pylori* plus MNU-treatment. The mice were inoculated with the *H. pylori* (*SS1* strain) three times a week for 4 weeks. The infected or no-infected mice were administered with MNU-containing drinking water repeated weekly for 8 weeks. The control group was administered with distilled water that did not contain MNU or *SSI*. Mice were sacrificed 8 months after MNU treatment, and the stomachs were obtained and histologically examined. *H. pylori* colonization in the mouse stomach was confirmed by rapid urease test.

### Statistical analysis

Sample size was predetermined based on the variability observed in preliminary and similar experiments. All mice were randomly assigned to experimental groups. Data were excluded from the analysis if an animal died during experiments. Investigators were not blinded to group allocation or outcome assessment. For all groups that were statistically compared, the variance between the groups was very similar. Statistical data analysis was performed using the two-tailed Student’s *t* test and one-way analysis of variance and data was expressed as mean ± standard deviation (SD) of three separate experiments. *P* values of <0.05 were considered statistically significant.

## Supplementary information

Supplemental figure legend

Supplementary Figure 1

Supplementary Figure 2

Supplementary Figure 3

Supplementary Figure 4

Supplementary Figure 5

Supplementary Figure 6

Supplementary Table 1

Supplementary Table 2

Supplementary Table 3

## References

[1] Bray F (2018). Global cancer statistics 2018: GLOBOCAN estimates of incidence and mortality worldwide for 36 cancers in 185 countries. CA Cancer J. Clin..

[2] Chen W (2016). Cancer statistics in China, 2015. CA Cancer J. Clin..

[3] Padmanabhan N, Ushijima T, Tan P (2017). How to stomach an epigenetic insult: the gastric cancer epigenome. Nat. Rev. Gastroenterol. Hepatol..

[4] Yamashita S (2018). Genetic and epigenetic alterations in normal tissues have differential impacts on cancer risk among tissues. Proc. Natl Acad. Sci. USA.

[5] Muratani M (2014). Nanoscale chromatin profiling of gastric adenocarcinoma reveals cancer-associated cryptic promoters and somatically acquired regulatory elements. Nat. Commun..

[6] Qamra A (2017). Epigenomic promoter alterations amplify gene isoform and immunogenic diversity in gastric adenocarcinoma. Cancer Discov..

[7] Grosse Y (2006). Carcinogenicity of nitrate, nitrite, and cyanobacterial peptide toxins. Lancet Oncol..

[8] Loh YH (2011). N-Nitroso compounds and cancer incidence: the European Prospective Investigation into Cancer and Nutrition (EPIC)-Norfolk Study. Am. J. Clin. Nutr..

[9] Hebels DG, Briede JJ, Khampang R, Kleinjans JC, de Kok TM (2010). Radical mechanisms in nitrosamine- and nitrosamide-induced whole-genome gene expression modulations in Caco-2 cells. Toxicol. Sci..

[10] Sakamoto K (2010). Inhibitor of kappaB kinase beta regulates gastric carcinogenesis via interleukin-1alpha expression. Gastroenterology.

[11] Hayakawa Y (2011). Apoptosis signal-regulating kinase 1 and cyclin D1 compose a positive feedback loop contributing to tumor growth in gastric cancer. Proc. Natl Acad. Sci. USA.

[12] Wang G, Yu Y, Chen X, Xie H (2001). Low concentration N-methyl-N’-nitro-N-nitrosoguanidine activates DNA polymerase-beta expression via cyclic-AMP-protein kinase A-cAMP response element binding protein pathway. Mutat. Res..

[13] Gao Z, Yang J, Huang Y, Yu Y (2005). N-methyl-N’-nitro-N-nitrosoguanidine interferes with the epidermal growth factor receptor-mediated signaling pathway. Mutat. Res..

[14] Gong C (2016). ATR-CHK1-E2F3 signaling transactivates human ribonucleotide reductase small subunit M2 for DNA repair induced by the chemical carcinogen MNNG. Biochim. Biophys. Acta.

[15] Shen J, Zhu H, Xiang X, Yu Y (2009). Differential nuclear proteomes in response to N-methyl-N’-nitro-N-nitrosoguanidine exposure. J. Proteome Res..

[16] Amieva M, Peek RM (2016). Pathobiology of Helicobacter pylori-Induced Gastric Cancer. Gastroenterology.

[17] Chen K (2016). Biphasic reduction of histone H3 phosphorylation in response to N-nitroso compounds induced DNA damage. Biochim. Biophys. Acta.

[18] Chai EZ (2016). Targeting transcription factor STAT3 for cancer prevention and therapy. Pharmacol. Ther..

[19] Pesic M, Greten FR (2016). Inflammation and cancer: tissue regeneration gone awry. Curr. Opin. Cell Biol..

[20] Yu H, Lee H, Herrmann A, Buettner R, Jove R (2014). Revisiting STAT3 signalling in cancer: new and unexpected biological functions. Nat. Rev. Cancer.

[21] Zhang Q (2006). STAT3 induces transcription of the DNA methyltransferase 1 gene (DNMT1) in malignant T lymphocytes. Blood.

[22] Przanowski P (2014). The signal transducers Stat1 and Stat3 and their novel target Jmjd3 drive the expression of inflammatory genes in microglia. J. Mol. Med..

[23] Pan YM (2016). STAT3 signaling drives EZH2 transcriptional activation and mediates poor prognosis in gastric cancer. Mol. Cancer.

[24] Lee H (2012). Acetylated STAT3 is crucial for methylation of tumor-suppressor gene promoters and inhibition by resveratrol results in demethylation. Proc. Natl Acad. Sci. USA.

[25] Kim E (2013). Phosphorylation of EZH2 activates STAT3 signaling via STAT3 methylation and promotes tumorigenicity of glioblastoma stem-like cells. Cancer Cell.

[26] Dasgupta M, Dermawan JK, Willard B, Stark GR (2015). STAT3-driven transcription depends upon the dimethylation of K49 by EZH2. Proc. Natl Acad. Sci. USA.

[27] Hossain DM (2013). FoxP3 acts as a cotranscription factor with STAT3 in tumor-induced regulatory T cells. Immunity.

[28] Nam KT (2004). The selective cyclooxygenase-2 inhibitor nimesulide prevents Helicobacter pylori-associated gastric cancer development in a mouse model. Clin. Cancer Res..

[29] Nagashima H (2015). Toll-like receptor 10 in Helicobacter pylori Infection. J. Infect. Dis..

[30] Vermeulen L, De Wilde G, Van Damme P, Vanden Berghe W, Haegeman G (2003). Transcriptional activation of the NF-kappaB p65 subunit by mitogen- and stress-activated protein kinase-1 (MSK1). EMBO J..

[31] Yuan J, Zhang F, Niu R (2015). Multiple regulation pathways and pivotal biological functions of STAT3 in cancer. Sci. Rep..

[32] Reyes D (2014). Activation of mitogen- and stress-activated kinase 1 is required for proliferation of breast cancer cells in response to estrogens or progestins. Oncogene.

[33] Josefowicz SZ (2016). Chromatin Kinases Act on transcription factors and histone tails in regulation of inducible transcription. Mol. Cell.

[34] Zhang Y, Liu G, Dong Z (2001). MSK1 and JNKs mediate phosphorylation of STAT3 in UVA-irradiated mouse epidermal JB6 cells. J. Biol. Chem..

[35] Cai W (2014). Genome-wide analysis of regulation of gene expression and H3K9me2 distribution by JIL-1 kinase mediated histone H3S10 phosphorylation in Drosophila. Nucleic Acids Res..

[36] Sawicka A (2014). H3S28 phosphorylation is a hallmark of the transcriptional response to cellular stress. Genome Res..

[37] Qin JJ (2014). NFAT as cancer target: mission possible?. Biochim. Biophys. Acta.

[38] Xiao Z. J., et al. NFATc2 enhances tumor-initiating phenotypes through the NFATc2/SOX2/ALDH axis in lung adenocarcinoma. *Elife* **6**, e26733 (2017). 10.7554/eLife.26733 PMC5570574 28737489

[39] Gerlach K (2012). Transcription factor NFATc2 controls the emergence of colon cancer associated with IL-6-dependent colitis. Cancer Res..

[40] Shen H, Laird PW (2013). Interplay between the cancer genome and epigenome. Cell.

[41] Feinberg AP, Koldobskiy MA, Gondor A (2016). Epigenetic modulators, modifiers and mediators in cancer aetiology and progression. Nat. Rev. Genet..

[42] Wingelhofer B (2018). Implications of STAT3 and STAT5 signaling on gene regulation and chromatin remodeling in hematopoietic cancer. Leukemia.

[43] Kim HG (2008). Mitogen- and stress-activated kinase 1-mediated histone H3 phosphorylation is crucial for cell transformation. Cancer Res..

[44] Li B (2015). Mitogen- and stress-activated Kinase 1 mediates Epstein-Barr virus latent membrane protein 1-promoted cell transformation in nasopharyngeal carcinoma through its induction of Fra-1 and c-Jun genes. BMC Cancer.

[45] Gordon K (2014). Immortality, but not oncogenic transformation, of primary human cells leads to epigenetic reprogramming of DNA methylation and gene expression. Nucleic Acids Res..

[46] Muller MR, Rao A (2010). NFAT, immunity and cancer: a transcription factor comes of age. Nat. Rev. Immunol..

[47] Perotti V (2016). NFATc2 is an intrinsic regulator of melanoma dedifferentiation. Oncogene.

[48] Baumgart S (2012). Restricted heterochromatin formation links NFATc2 repressor activity with growth promotion in pancreatic cancer. Gastroenterology.

[49] Foldynova-Trantirkova S (2010). Breast cancer-specific mutations in CK1epsilon inhibit Wnt/beta-catenin and activate the Wnt/Rac1/JNK and NFAT pathways to decrease cell adhesion and promote cell migration. Breast Cancer Res..

[50] Grivennikov S (2009). IL-6 and Stat3 are required for survival of intestinal epithelial cells and development of colitis-associated cancer. Cancer Cell.

[51] Sakamoto K, Maeda S (2010). Targeting NF-kappaB for colorectal cancer. Expert Opin. Ther. Targets.

[52] Grossi V, Peserico A, Tezil T, Simone C (2014). p38alpha MAPK pathway: a key factor in colorectal cancer therapy and chemoresistance. World J. Gastroenterol..

[53] Gao SP (2007). Mutations in the EGFR kinase domain mediate STAT3 activation via IL-6 production in human lung adenocarcinomas. J. Clin. Invest..

[54] Shin SY, Choi C, Lee HG, Lim Y, Lee YH (2012). Transcriptional regulation of the interleukin-11 gene by oncogenic Ras. Carcinogenesis.

