# Supplementary material for: STAT3 activates MSK1-mediated histone H3 phosphorylation to promote NFAT signaling in gastric carcinogenesis
Source: Oncogenesis. 2020 Feb 10;9(2):15. doi: 10.1038/s41389-020-0195-2 (PMC7010763; doi:10.1038/s41389-020-0195-2)
Supplement: Supplementary file 1 — Supplemental figure legend [file 41389_2020_195_MOESM1_ESM.doc]

**Figure supplement 1.** (a) Table of tumor formation rate in carcinogen-treated mice. (b) Cell anchorage-independent growth in soft agar was performed with the normal human gastric epithelial GES-1 cells and NOC-treated GES-1 cells. (c) Colony formation assay and xenograft assay in nude mice for NOC-treated cells. (d) Histone H3 phosphorylation was analyzed by WB in GES-1 and NOC-treated cells. (e) Cell cycle analysis of GES-1 and NOC-treated cells by flow cytometry. (f) AZD1480 2 μM, Tocilizumab 10 μM or STAT3 siRNA knockdown were used to analyze the p-H3S10 level by WB in MNNG-treated cell. (g) STAT3 Y705 phosphorylation and p-H3S10 levels in different MNNG-induced cell subcolonies. The analyses were repeated three times, and the results were expressed as mean ± SD.

**Figure supplement 2.** (a) MSK2 and Aurora B protein levels in NOC-transformed cells detected by WB analysis. (b) RT-qPCR analysis of MSK1 mRNA expression after NOC treatment for once (1T1W) or twice (2T1W) a week. (c) p-H3S10 level in NOC-treated cells with MSK1 siRNA knockdown. (d) Colony formation analysis in MSK1 stably silenced in NOC-transformed cells. (e) Tumor weight of xenograft assay was analyzed after H89 treatment or MSK1 stably silenced in NOC-transformed cells. (f) p-H3S10P level was detected by WB in H89-treated xenograft tissues. (g) p-H3S10 level in the gastric cancer cells treated by H89. (h) Colony formation ability of gastric cancer cells treated by H89 at different concentrations. (i) The representative images of MSK1 expression in paired gastric cancer tissue by IHC. Scale bar: 200μm. The analyses were repeated three times, and the results were expressed as mean ± SD. *p<0.05, **p<0.01, ***p<0.001.

**Figure supplement 3.** (a) WB analysis of MSK1 protein level in STAT3 overexpressed GES-1 cells. (b) MSK1 mRNA was examined by RT-qPCR after MNNG (2 μM) or MNU (0.5 mM) treatment once a week with or without AG490 or stattic inhibition in GES-1 cells. *p < 0.05. (C) STAT3 Y727 phosphorylation level after H89 treatment in NOC-transformed cells. The analyses were repeated three times, and the results were expressed as mean ± SD.

**Figure supplement 4.** (a) KEGG analysis of differentially expressed genes in NOC-transformed cells. (b) Gene set enrichment plots of differentially expressed genes belonging to the IL-6/JAK/STAT3 pathway in NOC-transformed cells. *P* value is determined by GSEA software. (c) Tag density profile of p-H3S10 distribution of genes showing the increase in p-H3S10 modifications. (d) Genomic distribution of p-H3S10 binding categorized based on associated type of genomic region in NOC-transformed cells. (e) The expression of NFATc2 was detected by WB in GES-1 and GC cells (AGS, MKN45, SGC7901). (f) NFATc2 mRNA expression in AGS cells with H89 treatment, MSK1 or STAT3 knockdown. The analyses were repeated three times, and the results were expressed as mean ± SD. *p < 0.05.

**Figure supplement 5.** (a) IL-11 expression by PCR after NOC treatment at different times. The analyses were repeated three times, and the results were expressed as mean ± SD. *p<0.05, **p<0.01. (b) The representative images of NFATc2 and IL-6 expression in human gastric cancer tissues by IHC. Scale bar: 200 μm.

**Figure supplement 6.** (a) Tumor weight of xenograft assay in NOC-transformed cell (left) and gastric cancer cell MKN45 (right) following the inhibition of STAT3 or NFATc2 by AZD1480 or CSA, respectively. The results were expressed as mean ± SD. *p < 0.05. (B) STAT3, MSKs and NFATc2 expression in different tumor types from TCGA database and analyzed by GEPIA. ACC, Adrenocortical carcinoma; BLCA, Bladder Urothelial Carcinoma; BRCA, Breast invasive carcinoma; CESC, Cervical squamous cell carcinoma and endocervical adenocarcinoma; CHOL, Cholangio carcinoma; COAD, Colon adenocarcinoma; DLBC, Lymphoid Neoplasm Diffuse Large B-cell Lymphoma; ESCA, Esophageal carcinoma; GBM, Glioblastoma multiforme; HNSC, Head and Neck squamous cell carcinoma; KICH, Kidney Chromophobe; KIRC, Kidney renal clear cell carcinoma; KIRP, Kidney renal papillary cell carcinoma; LAML, Acute Myeloid Leukemia; LGG, Brain Lower Grade Glioma; LIHC, Liver hepatocellular carcinoma; LUAD, Lung adenocarcinoma; LUSC, Lung squamous cell carcinoma; OV, Ovarian serous cystadenocarcinoma; PAAD, Pancreatic adenocarcinoma; PCPG, Pheochromocytoma and Paraganglioma; PRAD, Prostate adenocarcinoma; READ, Rectum adenocarcinoma; SARC, Sarcoma; SKCM, Skin Cutaneous Melanoma; STAD, Stomach adenocarcinoma; TGCT, Testicular Germ Cell Tumors; THCA, Thyroid carcinoma; THYM, Thymoma; UCEC, Uterine Corpus Endometrial Carcinoma; UCS, Uterine Carcinosarcoma.
