# Supplementary figures and images for: STAT3 activates MSK1-mediated histone H3 phosphorylation to promote NFAT signaling in gastric carcinogenesis
Source: Oncogenesis. 2020 Feb 10;9(2):15. doi: 10.1038/s41389-020-0195-2 (PMC7010763; doi:10.1038/s41389-020-0195-2)

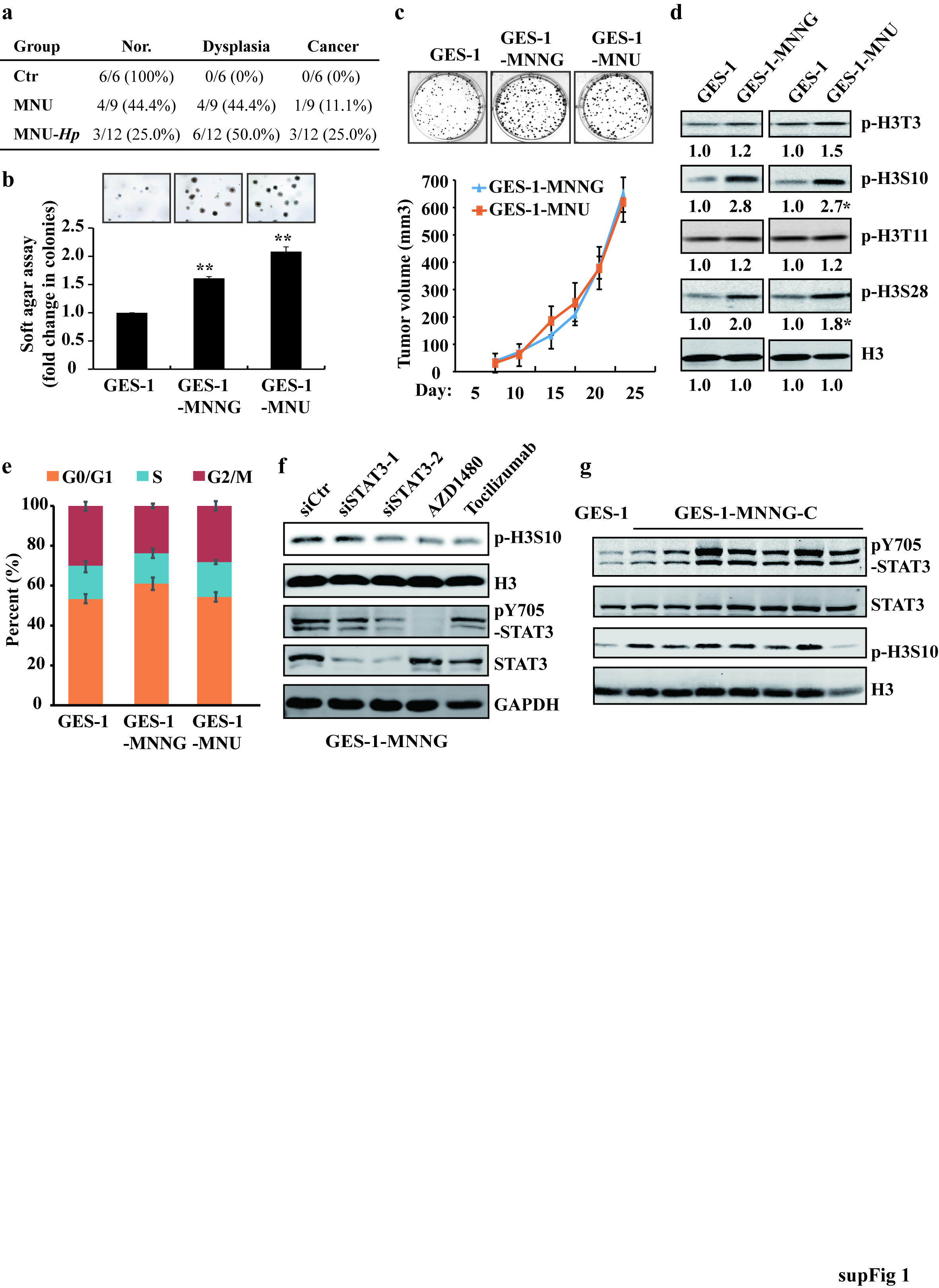

Supplement: Supplementary file 2 — Supplementary Figure 1 [file 41389_2020_195_MOESM2_ESM.tif]

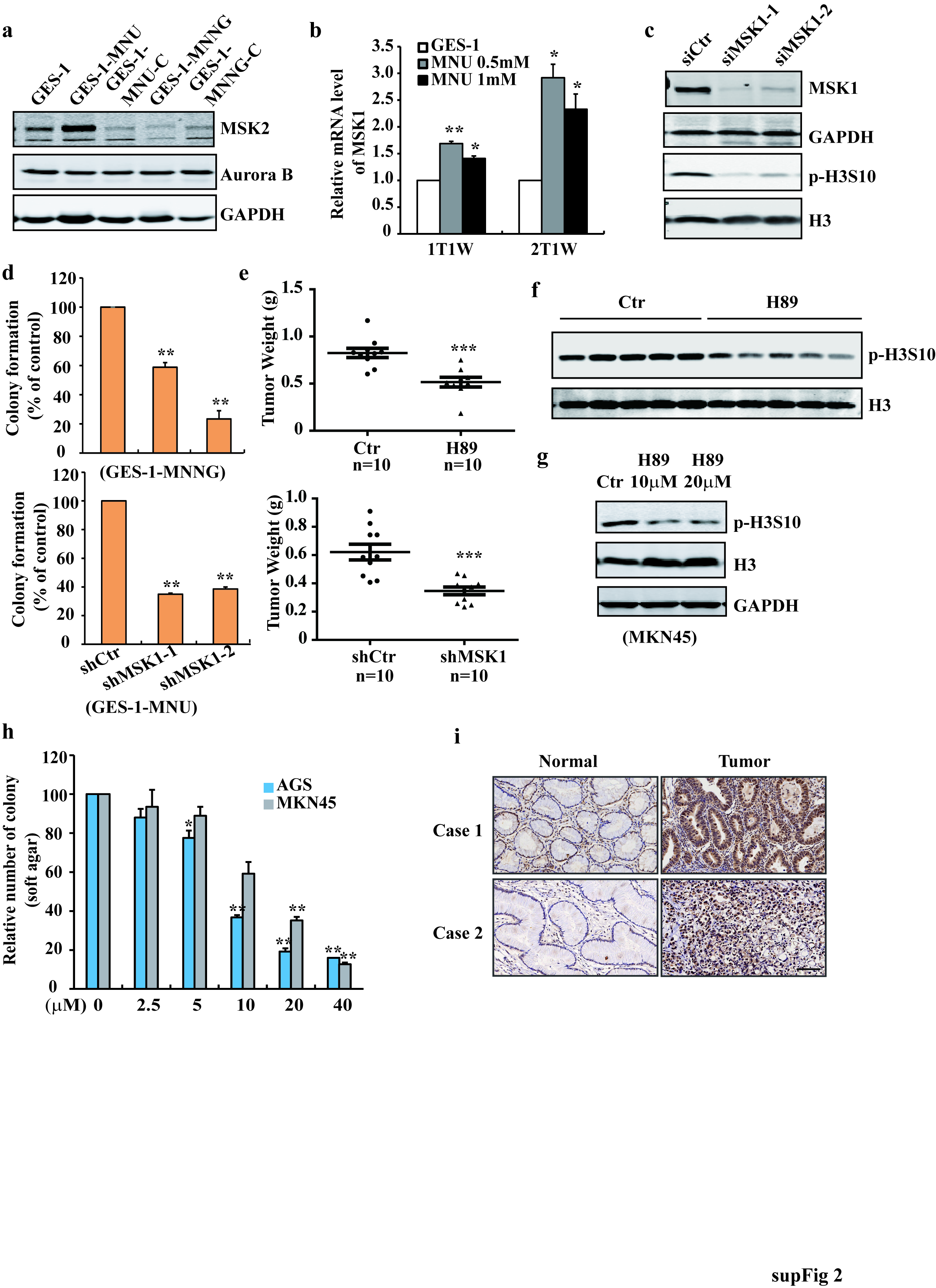

Supplement: Supplementary file 3 — Supplementary Figure 2 [file 41389_2020_195_MOESM3_ESM.tif]

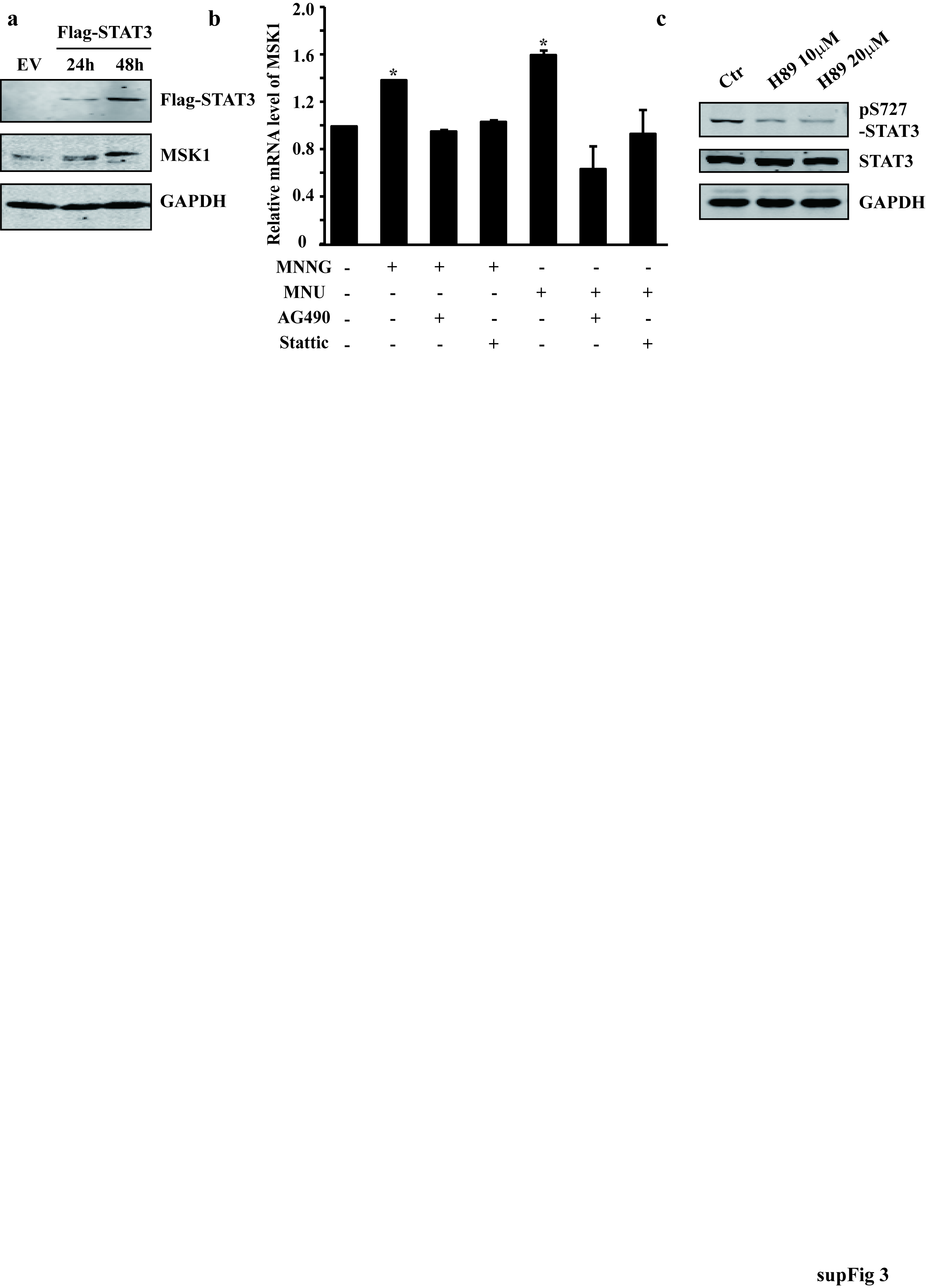

Supplement: Supplementary file 4 — Supplementary Figure 3 [file 41389_2020_195_MOESM4_ESM.tif]

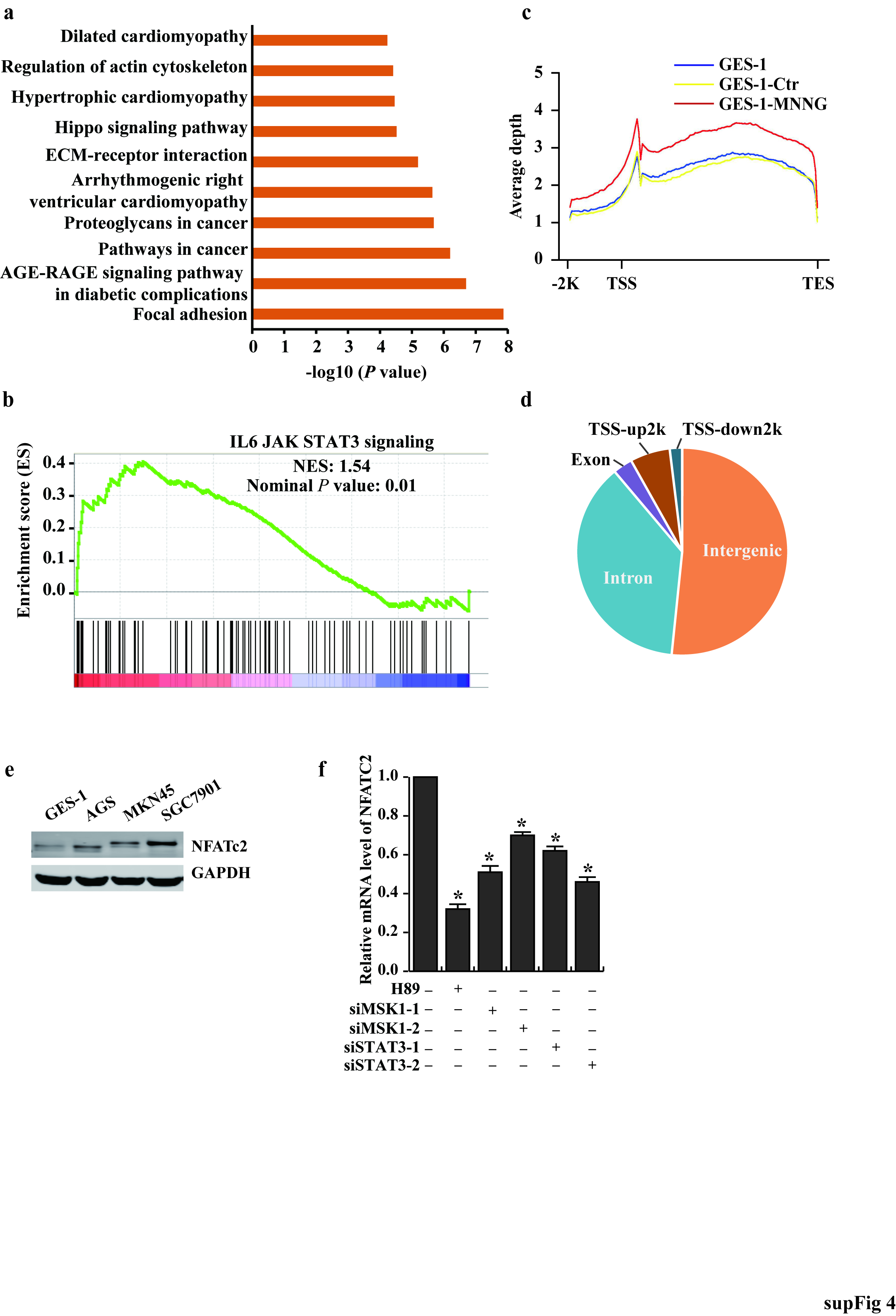

Supplement: Supplementary file 5 — Supplementary Figure 4 [file 41389_2020_195_MOESM5_ESM.tif]

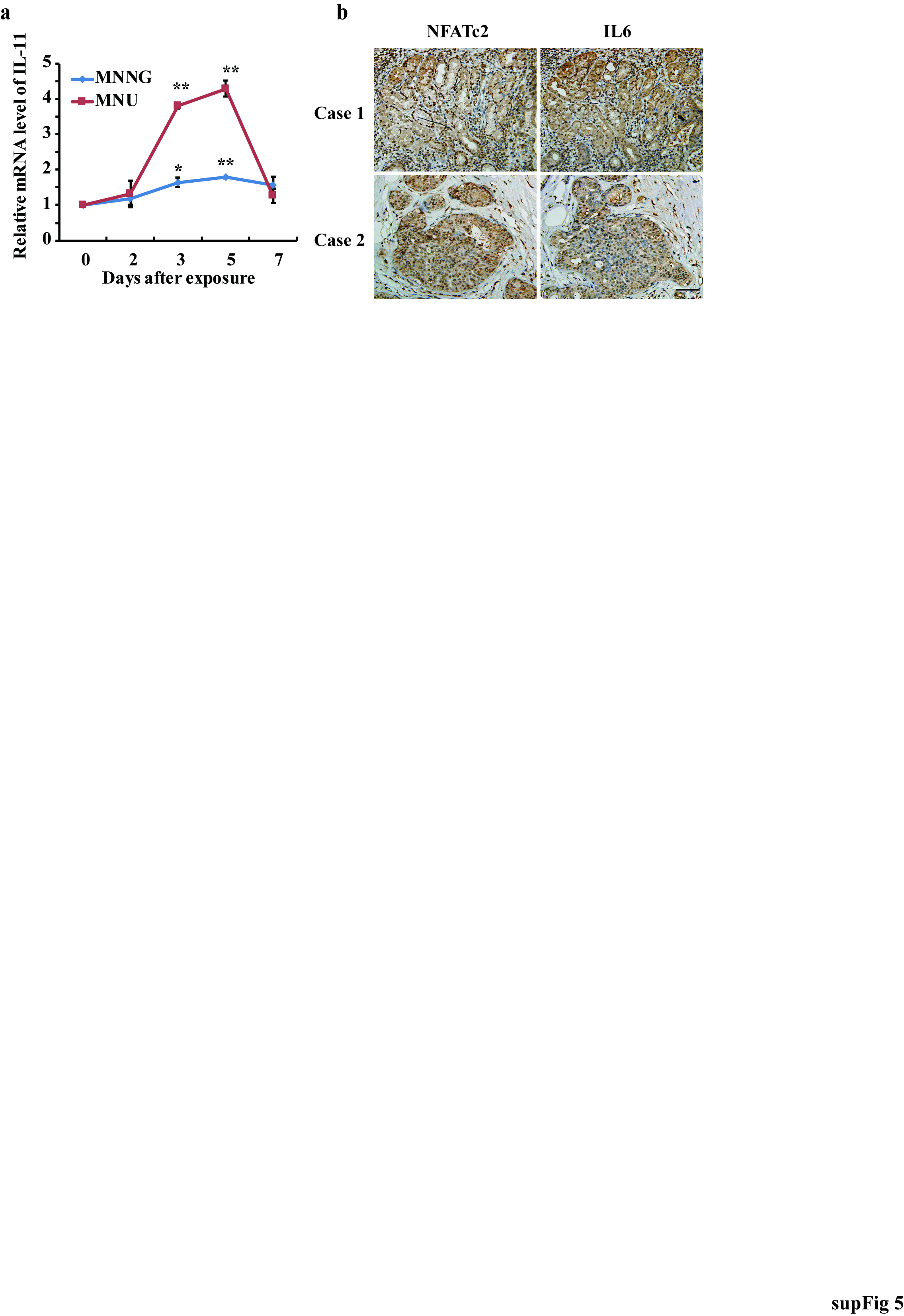

Supplement: Supplementary file 6 — Supplementary Figure 5 [file 41389_2020_195_MOESM6_ESM.tif]

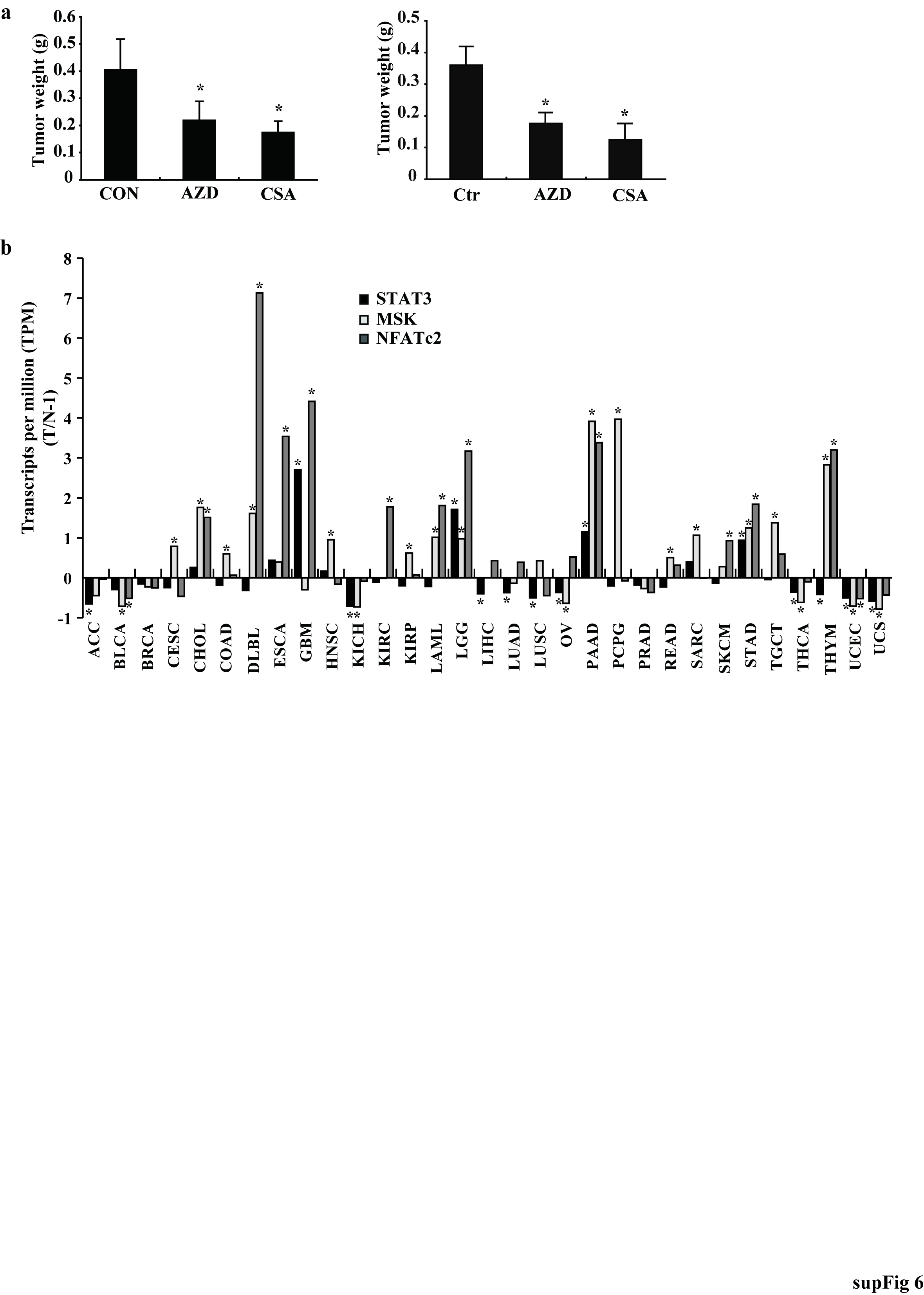

Supplement: Supplementary file 7 — Supplementary Figure 6 [file 41389_2020_195_MOESM7_ESM.tif]
