# Supplementary material for: STAT3 activates MSK1-mediated histone H3 phosphorylation to promote NFAT signaling in gastric carcinogenesis
Source: Oncogenesis. 2020 Feb 10;9(2):15. doi: 10.1038/s41389-020-0195-2 (PMC7010763; doi:10.1038/s41389-020-0195-2)
Supplement: Supplementary file 8 — Supplementary Table 1 [file 41389_2020_195_MOESM8_ESM.docx]

Supplementary Table 1. Thirty overlapped genes with increased expresion in RNA-seq and enriched p-H3S10 signals in ChIP-seq.

| **gene symbol** | **gene id** | **ChIP-seq sites (numbers)** | **RNA-seq GES-1 FPKM** | **RNA-seq GES-1-MNNG FPKM** | **RNA-seq log2 Ratio** | **Blast nr** |
| --- | --- | --- | --- | --- | --- | --- |
| MSI2 | 124540 | 26 | 14.5 | 30.74 | 1.084064 | gi\|635089799\|ref\|XP_008009553.1\|/2.79539e-148/PREDICTED: RNA-binding protein Musashi homolog 2 isoform X11 [Chlorocebus sabaeus] |
| RALGPS1 | 9649 | 22 | 1.45 | 3.75 | 1.370838 | gi\|635071085\|ref\|XP_008004377.1\|/0/PREDICTED: ras-specific guanine nucleotide-releasing factor RalGPS1 isoform X4 [Chlorocebus sabaeus] |
| NFATC2 | 4773 | 16 | 2.59 | 5.33 | 1.041183 | gi\|209862843\|ref\|NP_001129493.1\|/0/nuclear factor of activated T-cells, cytoplasmic 2 isoform D [Homo sapiens] |
| PPP2R2C | 5522 | 14 | 2.28 | 4.99 | 1.130006 | gi\|332801069\|ref\|NP_001193923.1\|/0/protein phosphatase 2, regulatory subunit B, gamma isoform c [Homo sapiens] |
| SLC22A23 | 63027 | 14 | 1.49 | 3.57 | 1.260612 | gi\|557129033\|ref\|NP_001273385.1\|/1.00307e-159/solute carrier family 22 member 23 isoform c [Homo sapiens] |
| ADAMTS16 | 170690 | 11 | 0.57 | 2.21 | 1.955013 | gi\|110735441\|ref\|NP_620687.2\|/0/A disintegrin and metalloproteinase with thrombospondin motifs 16 preproprotein [Homo sapiens] |
| PDZD2 | 23037 | 8 | 0.35 | 1.03 | 1.557218 | gi\|87196343\|ref\|NP_835260.2\|/0/PDZ domain-containing protein 2 [Homo sapiens] |
| ANGPTL4 | 51129 | 7 | 1.66 | 4.98 | 1.584963 | gi\|37181670\|gb\|AAQ88642.1\|/0/NL2 [Homo sapiens] |
| ARRB1 | 408 | 7 | 2.83 | 9.04 | 1.675521 | gi\|3493147\|gb\|AAC33295.1\|/0/beta-arrestin 1A [Homo sapiens] |
| ABCA4 | 24 | 7 | 0.06 | 0.95 | 3.984893 | gi\|105990541\|ref\|NP_000341.2\|/0/retinal-specific ATP-binding cassette transporter [Homo sapiens] |
| UNC5B | 219699 | 6 | 0.76 | 3.26 | 2.100801 | gi\|349585210\|ref\|NP_001231818.1\|/0/netrin receptor UNC5B isoform 2 precursor [Homo sapiens] |
| HIPK2 | 28996 | 5 | 1.72 | 3.48 | 1.016679 | gi\|164420691\|ref\|NP_001106710.1\|/0/homeodomain-interacting protein kinase 2 isoform 2 [Homo sapiens] |
| SLC9A8 | 23315 | 4 | 3.83 | 8.18 | 1.094756 | gi\|386781486\|ref\|NP_001247420.1\|/0/sodium/hydrogen exchanger 8 isoform 1 [Homo sapiens] |
| ATP9A | 10079 | 4 | 8.6 | 17.9 | 1.057551 | gi\|65301139\|ref\|NP_006036.1\|/0/probable phospholipid-transporting ATPase IIA [Homo sapiens] |
| C16orf62 | 57020 | 4 | 6.52 | 13.44 | 1.043589 | gi\|304766523\|ref\|NP_064710.4\|/0/UPF0505 protein C16orf62 isoform 1 [Homo sapiens] |
| EGFLAM | 133584 | 4 | 0.18 | 0.92 | 2.353637 | gi\|52545930\|emb\|CAH56137.1\|/8.77673e-110/hypothetical protein [Homo sapiens] |
| TK2 | 7084 | 3 | 1.65 | 6.49 | 1.975752 | gi\|119603423\|gb\|EAW83017.1\|/3.05575e-148/thymidine kinase 2, mitochondrial, isoform CRA_e [Homo sapiens] |
| CCDC92 | 80212 | 3 | 2.99 | 11.49 | 1.942161 | gi\|13376725\|ref\|NP_079416.1\|/7.34667e-151/coiled-coil domain-containing protein 92 isoform 1 [Homo sapiens] |
| ATXN1 | 6310 | 3 | 0.56 | 3.98 | 2.82927 | gi\|426351699\|ref\|XP_004043367.1\|/0/PREDICTED: ataxin-1 [Gorilla gorilla gorilla] |
| AMOTL1 | 154810 | 3 | 12.74 | 33.59 | 1.398667 | gi\|22027646\|ref\|NP_570899.1\|/0/angiomotin-like protein 1 isoform 1 [Homo sapiens] |
| ST8SIA2 | 8128 | 3 | 0.52 | 6.49 | 3.641635 | gi\|239740399\|gb\|ACS13735.1\|/0/alpha-2,8-sialyltransferase 8B [Homo sapiens] |
| FAM212B | 55924 | 3 | 0.77 | 2.24 | 1.540568 | gi\|39545575\|ref\|NP_945120.1\|/4.84673e-137/protein FAM212B isoform 2 [Homo sapiens] |
| NPTXR | 23467 | 3 | 2.46 | 9.85 | 2.001465 | gi\|119580677\|gb\|EAW60273.1\|/0/neuronal pentraxin receptor, isoform CRA_a [Homo sapiens] |
| TNFRSF1B | 7133 | 3 | 0.86 | 6.33 | 2.879797 | gi\|4507577\|ref\|NP_001057.1\|/0/tumor necrosis factor receptor superfamily member 1B precursor [Homo sapiens] |
| TRIM62 | 55223 | 3 | 2.51 | 5.47 | 1.123853 | gi\|767904952\|ref\|XP_011540007.1\|/0/PREDICTED: E3 ubiquitin-protein ligase TRIM62 isoform X1 [Homo sapiens] |
| ZBTB7C | 201501 | 3 | 0.15 | 2.39 | 3.993976 | gi\|918575127\|ref\|XP_013365990.1\|/0/PREDICTED: zinc finger and BTB domain-containing protein 7C isoform X2 [Chinchilla lanigera] |
| DNMT3B | 1789 | 2 | 5.36 | 14.88 | 1.47307 | gi\|333440489\|ref\|NP_001193985.1\|/0/DNA (cytosine-5)-methyltransferase 3B isoform 8 [Homo sapiens] |
| NALCN | 259232 | 2 | 1.43 | 3.72 | 1.379287 | gi\|767978823\|ref\|XP_011519369.1\|/0/PREDICTED: sodium leak channel non-selective protein isoform X2 [Homo sapiens] |
| SORBS2 | 8470 | 2 | 0.03 | 0.72 | 4.584963 | gi\|224586853\|ref\|NP_001139146.1\|/0/sorbin and SH3 domain-containing protein 2 isoform 7 [Homo sapiens] |
| TMEM217 | 221468 | 2 | 0.21 | 2.29 | 3.446886 | gi\|767939425\|ref\|XP_011512669.1\|/9.43751e-113/PREDICTED: transmembrane protein 217 isoform X3 [Homo sapiens] |
