# Supplementary material for: STAT3 activates MSK1-mediated histone H3 phosphorylation to promote NFAT signaling in gastric carcinogenesis
Source: Oncogenesis. 2020 Feb 10;9(2):15. doi: 10.1038/s41389-020-0195-2 (PMC7010763; doi:10.1038/s41389-020-0195-2)
Supplement: Supplementary file 9 — Supplementary Table 2 [file 41389_2020_195_MOESM9_ESM.doc]

Supplementary Table 2. Primer list

| Primers | Sequence |
| --- | --- |
| MSK1 forward | 5’-TTCCTTTGTTGCTCCTTCCATC-3’ |
| MSK1 reverse | 5’-CAACATTTGTCACTCCAGGACG-3’ |
| MSK2 forward | 5’-CCTTCCCTGCAACTCTATCTGG-3’, |
| MSK2 reverse | 5’-GGACTGTCCTTTCCTCTCCTACC-3’ |
| NFATc2 forward | 5’-GAGGGGCTGTCAAAGCTCC-3’ |
| NFATc2 reverse | 5’-ACAGTTTTCCCCGTGATTCGG-3’ |
| IL-6 forward | 5’-ACTCACCTCTTCAGAACGAATTG-3’ |
| IL-6 reverse | 5’-AGCCATCTTTGGAAGGTTCAG-3’ |
| IL-11 forward | 5’-TGACGGAGATCACAGTCTGG-3’ |
| IL-11 reverse | 5’-CTTTAGGGAAGGACCACCTG-3’ |
| STAT3 forward | 5’-CCAAGGAGGAGGCATTCG-3’ |
| STAT3 reverse | 5’-ACATCGGCAGGTCAATGG-3’ |
| MSK1 promoter  STAT3-control forward | 5’-AGCAAATAATCCCATCTAAC-3’ |
| MSK1 promoter  STAT3-control reverse | 5’-ACTGGGAGGACTCTGAAC-3’ |
| MSK1 promoter  STAT3-BS1 forward | 5’-GTAGGCAGTCATGGTTTAGTGTG-3’, |
| MSK1 promoter  STAT3-BS1 reverse | 5’-TAGCTCAAGGGCAGTTAAAAAAC-3’ |
| MSK1 promoter  STAT3-BS2 forward | 5’-TTGGTTATCTCAAATGCCTCC-3’ |
| MSK1 promoter  STAT3-BS2 reverse | 5’-GAGAATCTCGCACGGATG-3’ |
| NFATc2 promoter control forward | 5’-TGGTCACCCTGTTCTTGCTTC-3’ |
| NFATc2 promoter control reverse | 5’-GGTGGCTTGCCTGTTGATTTAC-3’ |
| NFATc2 promoter  (p-H3S10 enriched sites) forward | 5’-GGAGTCTCGGGCTTGATTTATG-3’ |
| NFATc2 promoter  (p-H3S10 enriched sites) reverse | 5’-CGTGCGAGTGTCCAAGTCAG-3’ |
| NFATc2 intron 1 (H3S10 enriched sites) forward | 5’-CTCCCATGCTGTGAGATGACTG-3’ |
| NFATc2 intron 1 (H3S10 enriched sites) reverse | 5’-TACCCTTGCCCAGAGCTGAAC-3’ |
| NFATc2 intron 2 (H3S10 enriched sites) forward | 5’-GGGCATTTCTGGTCTGTCATTTAT-3’ |
| NFATc2 intron 2 (H3S10 enriched sites) reverse | 5’-CAGAACCTTCCCTCAACCCTC-3’ |
| NFATc2 promoter  STAT3-BS1 forward | 5’-CCAGCATCTATCTCCAGCAGG-3’ |
| NFATc2 promoter  STAT3-BS1 reverse | 5’-GACTTGAACTTAAAGAGGCATCG-3’ |
| NFATc2 promoter  STAT3-BS2 forward | 5’-GACTTTTCTCCCGAACAATCAG-3’ |
| NFATc2 promoter  STAT3-BS2 reverse | 5’-CCGTCCATCTCCAAAACTAAGG-3’ |
