# Supplementary material for: STAT3 activates MSK1-mediated histone H3 phosphorylation to promote NFAT signaling in gastric carcinogenesis
Source: Oncogenesis. 2020 Feb 10;9(2):15. doi: 10.1038/s41389-020-0195-2 (PMC7010763; doi:10.1038/s41389-020-0195-2)
Supplement: Supplementary file 10 — Supplementary Table 3 [file 41389_2020_195_MOESM10_ESM.doc]

Supplementary Table 3. siRNAs and shRNA list

| Name | Sequence |
| --- | --- |
| Control siRNA | 5’-CUUACGCUGAGUACUUCGATT-3’ |
| STAT3 siRNA1 | 5’-GAGGAUCCCGGAAAUUUAA-3’ |
| STAT3 siRNA2 | 5’-GAGUUGAAUUAUCAGCUUATT-3’ |
| MSK1 siRNA1 | 5’-AGCAACCTTCCACGCCTTTAA-3’ |
| MSK1 siRNA2 | 5’-AGACCUAAUUCAGCGUCUUU-3’ |
| NFATc2 siRNA1 | 5’-ccauuaaacaggagcagaatt-3’ |
| NFATc2 siRNA2 | 5’-UCUACGUCAUCAAUGGGAAGAGAAA-3’ |
| MSK1 shRNA1 | GATCCGCAGATTTATGTTGGAGAGATCTCGAGATCTCTCCAACATAAATCTGCTTTTTG |
| MSK1 shRNA2 | GATCCGCACCATTTAAGCCAGTCATTCTCGAGAATGACTGGCTTAAATGGTGCTTTTTG |
